# Caspase-1 and IL-1β Processing in a Teleost Fish

**DOI:** 10.1371/journal.pone.0050450

**Published:** 2012-11-30

**Authors:** Marta I. R. Reis, Ana do Vale, Pedro J. B. Pereira, Jorge E. Azevedo, Nuno M. S. dos Santos

**Affiliations:** 1 Fish Immunology and Vaccinology, Instituto de Biologia Molecular e Celular (IBMC), Universidade do Porto, Porto, Portugal; 2 Instituto de Ciências Biomédicas Abel Salazar (ICBAS), Universidade do Porto, Porto, Portugal; 3 Biomolecular Structure, Instituto de Biologia Molecular e Celular (IBMC), Universidade do Porto, Porto, Portugal; 4 Organelle Biogenesis and Function, Instituto de Biologia Molecular e Celular (IBMC), Universidade do Porto, Porto, Portugal; INRA, France

## Abstract

Interleukine-1β (IL-1β) is the most studied pro-inflammatory cytokine, playing a central role in the generation of systemic and local responses to infection, injury, and immunological challenges. In mammals, IL-1β is synthesized as an inactive 31 kDa precursor that is cleaved by caspase-1 generating a 17.5 kDa secreted active mature form. The caspase-1 cleavage site strictly conserved in all mammalian IL-1β sequences is absent in IL-1β sequences reported for non-mammalian vertebrates. Recently, fish caspase-1 orthologues have been identified in sea bass (*Dicentrarchus labrax*) and sea bream (*Sparus aurata*) but very little is known regarding their processing and activity. In this work it is shown that sea bass caspase-1 auto-processing is similar to that of the human enzyme, resulting in active p24/p10 and p20/p10 heterodimers. Moreover, the presence of alternatively spliced variants of caspase-1 in sea bass is reported. The existence of caspase-1 isoforms in fish and in mammals suggests that they have been evolutionarily maintained and therefore are likely to play a regulatory role in the inflammatory response, as shown for other caspases. Finally, it is shown that sea bass and avian IL-1β are specifically cleaved by caspase-1 at different but phylogenetically conserved aspartates, distinct from the cleavage site of mammalian IL-1β.

## Introduction

IL-1β is the most studied pro-inflammatory cytokine, much due to its role in mediating auto-inflammatory diseases (see e.g., [1], [2], [3], [4]). It is mainly produced by activated macrophages, monocytes, and dendritic cells and affects almost every cell type, playing a central role in the generation of systemic and local responses to infection, injury, and immunological challenges, although it can also have detrimental effects (see e.g., [5], [6], [1], [2], [3], [4]). IL-1β exerts its activity by binding to IL-1 type I receptor (IL-1RI), which then recruits IL-1 receptor accessory protein (IL-1RAP) forming a complex that triggers a series of phosphorylation events leading to the activation of IκB kinase (IKK) and MAPK pathways (see e.g., [7], [4], [8]). These signaling pathways activate in turn the transcription factors NF-κB and AP-1, resulting in the induction of genes encoding chemokines, cytokines, acute-phase proteins, cell adhesion molecules, and enzymes involved in the production of small pro-inflammatory substances [5]. In mammals, IL-1β is synthesized as an inactive 31 kDa signal peptide-less precursor molecule (proIL-1β) [9], [10]. In humans and mice, caspase-1, also known as ICE (interleukin-1β converting enzyme) [11], [12], [13], specifically cleaves proIL-1β after aspartate 116 and 117, respectively, yielding a C-terminal 17.5 kDa secreted active form [14], [15], [11], [12], [16], [17], [18]. Non-caspase-1-mediated cleavage mechanisms generating different active forms of IL-1β have also been described (reviewed in [2], [19], [4], [5]).

IL-1β has been identified in several fish species [20], [21], [22], [23], [24], [25], [26], [27], [28], [29], [30]. However, although it was reported that processing of IL-1β by a gilthead sea bream fibroblast cell line is abrogated by a specific caspase-1 inhibitor [31], neither direct evidence for the participation of caspase-1 in this processing event was provided nor the cleavage site in pro IL-1β was defined. This last issue is particularly important because the caspase-1 cleavage site reported for mammalian proIL-1β is absent in the proIL-1β sequences of non-mammalian vertebrates. Putative trout [32], sea bass [33], and chicken [34], [35], [36] recombinant mature IL-1β starting respectively at Ala^95^, Ala^86^, and Ala^106^, equivalent sequence-wise to the N-terminal residue of mature mammalian IL-1β, were shown to be biologically active. However, in none of these is the putative mature N-terminal preceded by an aspartate residue, making those sites rather improbable caspase-1 cleavage sequences. Furthermore, it was shown that in trout a 29 kDa IL-1β precursor expressed in RTS-11 cells was processed to a 24 kDa peptide [37], whereas a 15 kDa band has been detected by Western blotting with an anti- IL-1β antibody of phytohemagglutinin activated carp leucocyte culture supernatant [38], but the mechanism and exact cleavage site of processing were not clarified [39], [40], [41].

Caspase-1 is the prototype of a family of inflammatory caspases that contain a caspase recruitment domain (CARD) within their amino-terminal prodomains (see e.g., [5], [42]). Normally, it exists in the cytoplasm as a 45 kDa inactive precursor [43], being activated by CARD oligomerization in a multiprotein complex known as the inflammasome (see e.g., [44], [45], [46], [47], [48], [49], [50], [51]). Once activated, caspase-1 processes the pro-inflammatory cytokines starting an inflammatory response. Caspase-1 was recently found to be also involved in pyroptosis, a rapid active cell death programme characterized by early plasma-membrane rupture and release of proinflammatory intracellular contents ([52]; reviewed in [53], [54]). In humans, caspase-1 is activated by cleavage after Asp^103^, D^119^, D^297^ and D^316^, releasing the 11.5 kDa prodomain and a 2 kDa linker peptide and originating the active heterodimer p20/p10 [17], [55], [18], [56], [57].

Fish inflammatory caspases have been primarily sequenced in zebrafish [58] and sea bass (GenBank accession no.: DQ198377), the later being a caspase-1 homologue that has recently also been reported for sea bream [59], but there is still no functional data from these enzymes. Here, the characterization of the sea bass caspase-1 homologue, its processing and activity are reported. It is shown that sea bass caspase-1 auto-processing follows the mechanism described for the mammalian enzyme, yielding p24/p10 and p20/10 active heterodimers. In addition, three other caspase-1 isoforms have been identified. Finally, analysis of sea bass IL-1β processing by active sea bass caspase-1 revealed that the cleavage of the cytokine occurs at a phylogenetically conserved aspartate residue, distinct from the cleavage site in birds.

## Materials and Methods

### Ethics Statement

This study was carried out in accordance with European and Portuguese legislation for the use of animals for scientific purposes (Directive 86/609/EEC; Decreto-Lei 129/92; Portaria 1005/92). The work was approved by Direcção Geral de Veterinária, the Portuguese authority for animal protection.

### Fish

Sea bass (*Dicentrarchus labrax*) were kept in a recirculating, ozone-treated salt-water (25–30‰) system at 20±1°C, and fed at a ratio of 2% body weight per day. For organ collection fish were euthanized with 2-phenoxyethanol (Panreac; >5 ml/10 L).

### Cloning and sequencing of caspase-1

Messenger RNA was purified with the MicroPoly(A)Pure^TM^ kit (Ambion®) and transcribed to cDNA using the Superscript^TM^ II First-Strand Synthesis System (Invitrogen). PCRs were done according to standard procedures. For amplifying full length sequences Pfu DNA polymerase was used in the reactions. DNA sequencing was performed using primers detailed in Table S1. Complementary DNAs used for 5′ Rapid Amplification of cDNA Ends (5′RACE) experiments were purified using High Pure PCR Product Purification Kit reagents (Roche), and used with the 5′RACE System from Invitrogen (Version 2.0). The purified cDNA was dATP tailed using Recombinant Terminal Transferase (Roche).

Messenger RNA (∼340 ng) extracted from the head kidney of a fish 1 h after stimulation with LPS was reverse transcribed using primer APv (Table S1). Degenerate primers were designed based on conserved regions of caspase-1 amino acid sequences from different vertebrates. The cDNA was first PCR amplified using primers CASP1FW1/CASP1RV1 followed by a semi-nested amplification using primers CASP1FW1/CASP1RV2 (Table S1). A third amplification with primers CASP1FW1/CASP1RV2 was carried out and a PCR product with the expected size (168 bp) was purified, cloned and sequenced. This sequence was used to design the specific reverse primers DLCASP1RV1, DLCASP1RV2 and DLCASP1RV3 (Table S1), in order to obtain the 5′ untranslated region (5′-UTR) using the 5′RACE strategy. The obtained PCR product was purified, cloned and sequenced and, because it did not include the start codon, the 5′RACE strategy was repeated using again the specific reverse primers DLCASP1RV1 and DLCASP1RV2, plus the reverse primer DLCASP1RV5, designed based on the sequence obtained from the previous 5′RACE (Table S1). The fragment obtained was cloned, sequenced and used to design the specific primer DLCASP1FW7 (Table S1) in the 5′-UTR. This primer was combined with primer AUAP (Table S1), in a PCR aiming to obtain the complete sequence of sea bass caspase-1. Four PCR products ranging from 1500 to 1680 bp were purified, cloned into the pGEM®-T Easy Vector (Promega) and sequenced.

### Genomic DNA cloning and sequencing

Sea bass genomic DNA (gDNA) was isolated from erythrocytes of a single fish, as described by Stet and co-workers [60]. To obtain the complete caspase-1 gene, gDNA was amplified using the 5′-UTR primer DLCASP1FWEcoRI and the 3′-UTR primer DLCASP1RVEcoRI (Table S1), each containing a restriction site for EcoRI. The obtained PCR product was purified, digested with EcoRI (Fermentas) and cloned into the pET-32b vector (Novagen). Plasmid DNAs from three positive clones were purified and sequenced.

### Southern blotting

Restriction enzymes EcoRI, SpeI, BamHI, NcoI, NdeI, ApaLI (all zero cutters in sea bass caspase-1 gene), HindIII (single cutter in intron 1 of sea bass caspase-1 gene), and XbaI (double cutter in intron 1 of sea bass caspase-1 gene) were used to digest sea bass gDNA from 4 fish, according to the manufacturer's instructions (Fermentas). Digestion products were subjected to 0.8% agarose gel electrophoresis and Southern blotting [61].

A fragment of the sea bass caspase-1 coding region was amplified with primers DLCASP1FW4 and DLCASP1RV6 (Table S1). The PCR product (367 bp) was purified as previously described and labelled using the Gene Images^TM^ AlhPhos Direct^TM^ Labelling and Detection System (Amersham Biosciences). For signal generation and detection the Chemiluminescent Signal Generation and Detection with CDP-*Star* ^TM^ protocol from the same kit was followed.

### Sequence analysis of caspase-1

Full nucleotide and derived protein sequences from sea bass caspase-1 were compared with several caspase-1 sequences currently available at the GenBank database (www.ncbi.nlm.nih.gov). Multiple sequence alignments were made with CLUSTAL W [62]. Caspase-1 domains were based on PROSITE predictions (www.expasy.org/prosite). The Neighbour-joining phylogenetic tree was constructed using *MEGA* version 3.1 [63], using p-distance parameter and complete deletion of gaps. The phylogenetic tree was tested for reliability using 1000 bootstrap replications. The percentages of similarity and identity (Table S2) were calculated by pair-wise alignments by the program needle [64], with first and extending gap penalties of 10 and 0.5, respectively.

### Expression analysis of caspase-1 isoforms

Complementary DNA was synthesized from total RNA extracted from spleens of 9 non-stimulated fish as described above. The cDNAs were amplified using the forward primer DLCASP1FW11 (designed in an exon/intron boundary) for all isoforms and a reverse primer (Table S1) specific for different isoforms: isoform1, DLCASP1RV18 (between exon 7 e 8); isoform 2, DLCASP1RV16 (intron 7); isoform 3/4m, DLCASP1RV19 (between exon 6 and 8); and isoform 4, DLCASP1RV17 (intron 5). The reverse primer DLCASP1RV19 amplifies isoforms 3 and 4 but, in combination with the primer DLCASP1FW11, different size products are obtained (989 bp for isoform 3 and 1076 bp for isoform 4). The PCR products were purified and sequenced.

### Production of recombinant sea bass caspase-1 isoforms

The coding region of sea bass caspase-1 isoform 1 was amplified from the pGEM-T Easy plasmid DNA carrying the full length cDNA using specific primers DLCASP1FWNdeI and DLCASP1RVXhoI (Table S1). The PCR product was cloned into pGEM-T Easy, the plasmid DNA was digested with NdeI and XhoI (Fermentas), and the insert cloned into pET-28a (Novagen) in frame with N- and C-terminal His-tags. Recombinant sea bass caspase-1 was expressed in *E. coli* BL21 Rosetta (DE3) overnight at 37°C with 1 mM IPTG. The recombinant protein was extracted from bacterial cells as inclusion bodies and solubilised with 8 M Urea, 50 mM Tris-HCl, 0.2 M NaCl, 2 mM EDTA pH 8.0. The protein was refolded by dilution in 50 volumes of refolding buffer (50 mM Taps pH 8.5, 1.5 M Sorbitol, 1 mM TCEP, 24 mM NaCl and 1 mM KCl) overnight at 22°C. As a control, the refolding process was also performed in the presence of 100 µM of a specific caspase-1 inhibitor (Ac-YVAD-CHO, Caspase-1 inhibitor I, Calbiochem). The refolded protein was bound to an IMAC column (HisTrap HP, GE Healthcare) in refolding buffer. The column was washed with the same buffer supplemented with 10 mM imidazole and the protein was then eluted in 4 steps with increasing concentrations of imidazole (50, 100, 250 and 500 mM). The purified protein was analyzed by SDS-PAGE and, after blotted onto polyvinylidine difluoride membrane (PVDF), fragments of 24, 20 and 10 kDa were subjected to N-terminal Edman sequencing (Proteome Factory AG, Germany), in order to determine the cleavage sites between the large and small subunits of sea bass caspase-1.

The cDNAs encoding caspase-1 isoforms 2, 3 and 4 were obtained with the forward primer DLCASP1FWNdeI together with DLISO2/3RVXhoI for isoform 2 and 3 and with DLISO4RVXhoI for isoform 4 (Table S1). The cloning strategy, expression, purification and refolding protocols were those described for isoform 1, except that isoform 2 was cloned in pET-30a (Novagen).

### In vitro processing of caspase-1 isoforms

Two polyclonal antibodies directed against sea bass caspase-1 were produced (Davids Biotechnologie GmbH, Germany). An anti-p10 polyclonal antibody was raised against the peptide VHKEKDFISLLSST, and detected all caspase-1 forms containing the p10 domain. The antibody produced against the peptide QACRGNAGGAVLVSD corresponding to the carboxyl-terminal residues adjacent to the proteolytic cleavage site, detected processed forms that were cleaved at the p20 cleavage site (therefore, lacking the linker and the small subunit), but did not recognize unprocessed caspase-1.

Autoprocessing of the caspase-1 isoforms was analysed by SDS-PAGE and Western blotting. Samples of the isoforms collected after urea solubilisation or after an overnight refolding step in the presence or absence of 50–100 μM caspase-1 inhibitor were subjected to SDS-PAGE. Proteins in the gels were stained with Coomassie brilliant blue or were transferred to nitrocellulose membranes and probed with the rabbit anti-p10 or anti-p20 antibodies (both at a 1/10000 dilution) for 1 h at room temperature. Goat anti-rabbit Ig conjugated with alkaline phosphatase (Sigma) was used as the secondary antibody and the detection performed using 5-bromo-4-chloro-3-indolyl phosphate/nitro blue tetrazolium (BCIP/NBT).

The ability of active sea bass caspase-1 isoform 1 to process isoforms 2, 3 and 4 was tested by incubating 2 µg of the purified isoforms with recombinant active caspase-1 (p24/p10) at a ratio of 2∶1 (w/w) in a caspase-1 substrate buffer (0.1% v/w CHAPS, 0.1 M HEPES, 10 % v/w sucrose, 10 mM TCEP, pH 7.5) overnight at 22°C. As control, similar incubations were performed in the presence of 100 μM caspase-1 inhibitor. The proteins in the samples were precipitated with TCA and analysed by SDS-PAGE and Western blotting using the anti-p10 and anti-p20 antibodies, as described above, or transferred to a PVDF membrane for N-terminal Edman sequencing.

### Activity of recombinant sea bass caspase-1 isoforms

The activities of the refolded sea bass caspase-1 isoforms were tested by a fluorimetric assay using 0.1–2 µg of enzyme and a specific substrate for caspase-1 (Z-YVAD-AFC, Caspase-1 substrate VI, Calbiochem). To control cleavage specificity, 100 μM caspase-1 inhibitor was used. All assays were performed in duplicate. In each reaction, 71.5 μl of reaction buffer (0.2% CHAPS, 0.2 M HEPES, 20% sucrose, 29 mM DTT, pH 7.5) and 16 μM of caspase-1 substrate were used. The mixtures were incubated at 25°C and the fluorescence recorded for up to 3 h with 20 min intervals on a Spectra Max Gemini XS fluorimeter (Molecular Devices) at an excitation wavelength of 405 nm and an emission wavelength of 492 nm. The results were expressed as relative fluorescence units (RFU).

### In vitro production of sea bass, chicken and human IL-1β by rabbit reticulocytes lysates

#### Sea bass

Plasmids harboring different IL-1β forms have been produced as detailed below.

pET23proIL1β[His]: the coding region of sea bass proIL-1β was first amplified by PCR with primers DLIL1βFWNde3 and DLIL1βRVXho4 (Table S1) using a pGEX-4T-3 plasmid (GE Healthcare) carrying the proIL-1β cDNA (previously amplified from sea bass head kidney) as template [23]. The PCR product was purified and cloned into the pGEM-T Easy vector. After digestion with NdeI and XhoI, the sea bass proIL-1β was cloned into the expression vector pET-23a (Novagen) in frame with a C-terminal His-tag.

pET-23proIL1β, for producing proIL-1β: the histidine tag from pET-23proIL1β[His] was removed by site-directed mutagenesis (QuickChange® Site-Directed Mutagenesis Kit, Stratagene) using primers listed in Table S1.

pET-23proIL1β[D^60^A], pET-23proIL1β[D^100^A], pET-23proIL1β[D^252^A], and pET-23proIL1β[D^100^A/D^252^A], for producing mutated forms of proIL-1β in D^60^, D^100^, D^252^, or D^100^/D^252^: each aspartate was mutated to alanine by site-directed mutagenesis using pET-23proIL1β as template and primers listed in Table S1.

pET-23matureIL1β, for producing mature sea bass IL-1β (from S^101^ to Q^261^ and non-tagged): produced using pET-23proIL1β[His] as template and primers listed in Table S1.

pET-23matureIL1β[D^252^A], for producing mature sea bass IL-1β mutated at D^252^: aspartate 252 was mutated to alanine by site-directed mutagenesis using pET-23matureIL1β as template and primers listed in Table S1.

#### Chicken

The plasmids harboring the coding region of proIL-1β from chicken (GGpGEX-6P-1proIL1β), duck (APpET28proIL1β), goose (AApET28proIL1β) and turkey (MGpET28proIL1β) were kindly provided by Dr. Hsien-Sheng Yin and Dr. Long Huw Lee. The coding region of chicken proIL-1β was first amplified by PCR with primers listed in Table S1 using the plasmid GGpGEX-6P-1proIL1β as template. The PCR product was purified and cloned into the pGEM-T Easy vector. After digestion with NdeI and XhoI, the chicken proIL-1β was cloned into the expression vector pET-23a (Novagen) rendering GGpET23proIL1β.

GGpET23proIL1β[D^77^A], GGpET23proIL1β[D^80^A] and GGpET23proIL1β[D^82^A] for producing mutated forms of chicken proIL-1β in D^77^, D^80^ or D^82^: each aspartate was mutated to alanine by site-directed mutagenesis using GGpET23proIL1β as template and primers listed in Table S1.

GGpET23mature1IL1β, GG pET23mature2IL1β, and GGpET23matureIL1β for producing mature chicken IL-1β forms starting at I^119^, I^122^ and S^81^: produced using GGpET23proIL1β as template and primers listed in Table S1.

#### Human

HSpCMV-XL5proIL1β[D^116^A], HSpCMV-XL5proIL1β[D^128^A] and HSpCMV-XL5proIL1β[D^116^A/D^128^A], for producing mutated forms of human proIL-1β in D^116^, D^128^ or D^116^/D^128^: each aspartate was mutated to alanine by site-directed mutagenesis using HSpCMV-XL5proIL1β (IL-1β Human cDNA clone (NM_000576.2) in pCMV-XL5 plasmid from Origene (#SC122566)) as template and the primers listed in Table S1.

HSpET28mature1IL1β and HSpET28mature2IL1β, for producing mature human IL-1β from A^117^ and from S^129^: produced using HSpCMV-XL5proIL1β as template and primers described in Table S1.

The above mentioned plasmids, harboring different IL-1β forms, were used to synthesize the respective proteins using the TNT® T7 Quick Coupled Transcription/Translation kit (Promega) in the presence of [^35^S] methionine (specific activity >1000 Ci/mmol; PerkinElmer Life Sciences) following the manufacturer's instructions.

#### Production of recombinant sea bass IL-1β

His-tagged proIL-1β (proIL-1β[His]) was expressed in *E. coli* BL21 (DE3) CodonPlus at 37°C with 1 mM IPTG for 4 h, extracted from bacterial cells as inclusion bodies, solubilized as described for recombinant caspase-1 and purified under denaturing conditions using His-select^TM^ Nickel Affinity Gel (Sigma). The protein was then refolded by dialysis in three steps against 50 volumes of decreasing concentrations of urea (4 M for 12 h, 2 M for 12 h, 0 M overnight) in 10 mM sodium phosphate buffer pH 7.2 containing 184 mM NaCl at 4°C. The dialyzed protein was centrifuged at 12000 g and the supernatant analyzed by SDS- and Native-PAGE.

The cDNAs encoding mature IL-1β (starting at S^101^) with or without a C-terminal His-tag were amplified from pET23proIL1β[His] with the forward primer DLIL1BFWNde4 and the reverse primers DLIL1BRVXho4 or DLIL1BRVXho1, respectively (Table S1), cloned in pET-23a and expressed in *E. coli* BL21 (DE3) CodonPlus using the approach described for proIL-1β.

### In vitro processing of proIL-1β by caspase-1

The sea bass ^35^S-labeled IL-1β forms produced using rabbit reticulocyte lysates were incubated at 22°C for different periods with recombinant sea bass or human (Sigma) active caspase-1, with or without a caspase-1 substrate buffer (0.2% CHAPS, 0.2 M HEPES, 20% sucrose, 20 mM TCEP, pH 7.5) and in the presence or absence of 100µM of caspase-1 inhibitor. The same procedure was done for the human and chicken ^35^S-labeled IL-1β forms, but incubating overnight at 37 °C and with or without 250 µM of the caspase-1 inhibitor. The duck, turkey and goose ^35^S-labeled IL-1β forms were also incubated with recombinant active sea bass caspase-1 as described for chicken IL-1β. Samples were subjected to SDS-PAGE, and transferred to nitrocellulose membrane for autoradiography.

The ability of caspase-1 to process recombinant proIL-1β was tested at 22°C by mixing recombinant active caspase-1 with recombinant proIL-1β[His] at a ratio of 1∶2 (v/v) in the caspase-1 substrate buffer, in the presence or absence of 20 μM caspase-1 inhibitor. The samples were precipitated with TCA and subjected to SDS-PAGE. Proteins in the gels were stained with Coomassie brilliant blue for mass spectrometry (MS) analysis, or blotted onto a PVDF membrane for N-terminal Edman sequencing, or analyzed by Western blotting using rabbit anti-sea bass IL-1β or anti-His (Santa Cruz) antibodies. For antibody production, dialyzed recombinant proIL-1β[His] was further purified on a UNO^TM^ Q column (Bio-Rad) using 20 mM Tris pH 8.0 and 20 mM Tris pH 8.0 1.0 M NaCl as buffer system. The fractions containing proIL-1β[His] were pooled and subjected to SDS-PAGE. The band corresponding to proIL-1β[His] was excised from the gel and used to immunize rabbits (Davids Biotechnologie GmbH). This antibody recognizes proIL-1β and processed IL-1β fragments.

### Sea bass IL-1β processing in vivo

Over 20 fish were injected intraperitoneally with 200 µl of *Photobacterium damselae piscicida* concentrated heat-inactivated extracellular products, prepared as described elsewhere [65]. After 3 h, cells from the peritoneal cavity were collected by washing with 10 ml of HBSS, centrifuged and resuspended in fresh HBSS [66], [67]. Cells were counted, diluted to a final concentration of 1×10^6^ cells/ml and incubated for 3 h at 22°C in the presence or absence of 100 µM caspase-1 inhibitor. One milliliter of cell suspension was centrifuged at 400 g, the cell pellet resuspended in gel loading buffer and the proteins in the supernatant precipitated with TCA. After SDS-PAGE, samples were analyzed by Western blotting, using the rabbit anti-IL1β antibody, as described above.

## Results

### The sea bass caspase-1 transcript is alternatively spliced

**Figure 1:**
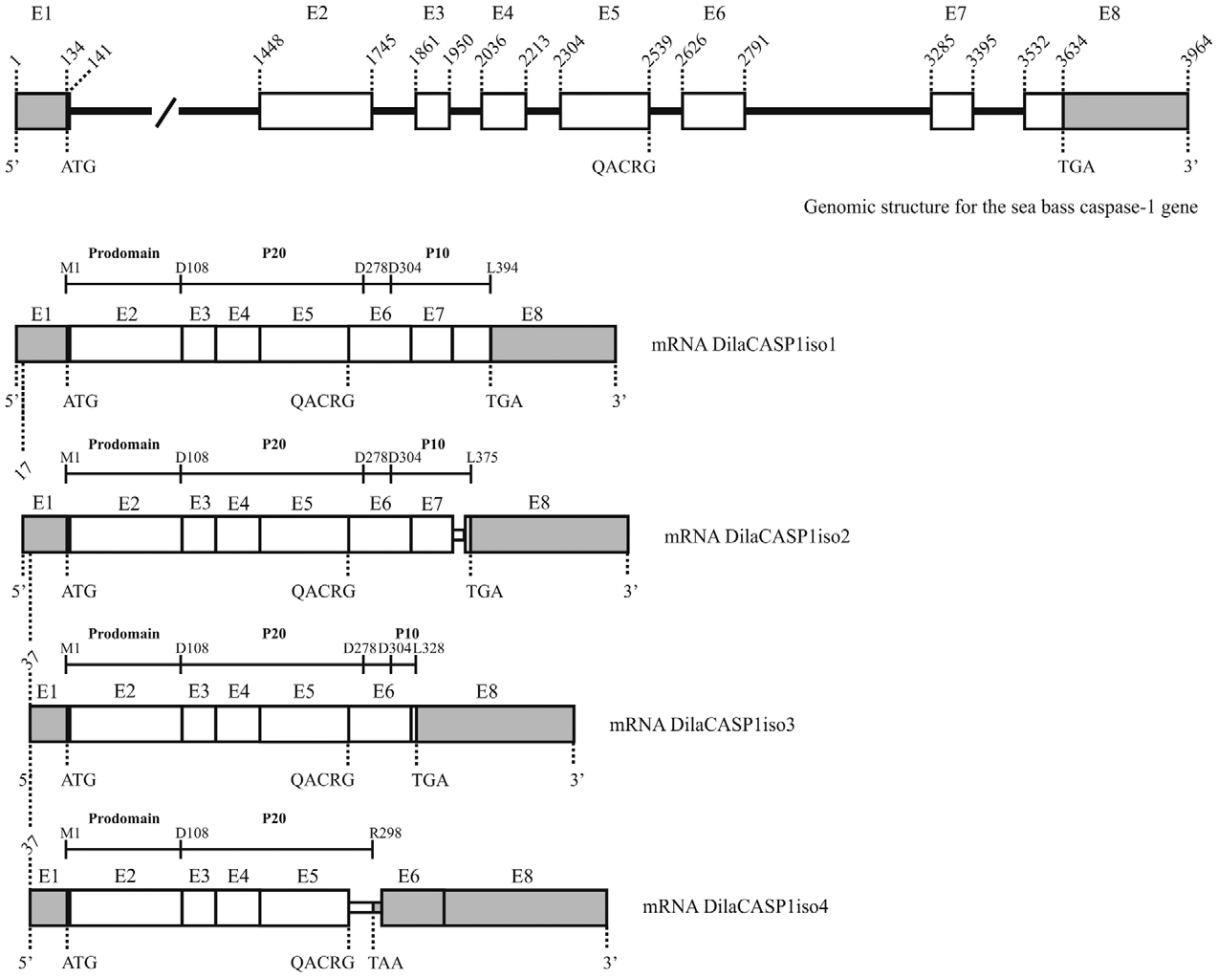
Schematic representation of the genomic organization and alternative splicing of sea bass caspase-1. The exons are represented by boxes (E1-E8) and introns by a solid line (introns larger than 800 bp are represented by an interrupted solid line). Coding and untranslated regions are indicated by white and shaded boxes, respectively. Nucleotide numbers are given above the boxes. Caspase-1 domains and cleavage sites are schematically represented on top of each transcript. ATG: starting codon; QACRG: caspase-1 pentapeptide active-site motif; TGA/TAA: stop codon.

The sea bass caspase-1 gene (GenBank accession number: DQ198377) was found to consist of 8 exons and 7 introns (Fig. 1), while the human caspase-1 gene has 10 exons and 9 introns (Ensembl transcript ID ENST00000260309) and presents much longer intronic regions (8266 bp *vs.* 2318 bp). Southern blot analysis suggested that, similar to the mouse and human caspase-1 genes [68], [69], [70], sea bass caspase-1 is a single copy gene (Fig. S1A), although four different transcripts encoding sea bass caspase-1 have been detected by RT-PCR using specific primers (Fig. 1, S1B and S1C). The corresponding deduced proteins are referred to as isoform 1–4 (DilaCASP1iso1-DilaCASP1iso4; GenBank accession numbers DQ198376, HQ398873, HQ398874 and HQ398875, respectively). DilaCASP1iso1 contains the typical caspase family p20 and p10 domain profiles and a caspase family active site signature (K^257^PKIIIIQACRG), as well as a characteristic CARD-containing domain. DilaCASP1iso2 and DilaCASP1iso3 have a shorter p10 domain, whereas DilaCASP1iso4 contains a longer p20 domain and no p10 (Fig. S1B). All isoforms comprise the active center pentapeptide QACRG.

Pair-wise alignments confirmed the homology of DilaCASP1iso1 with caspase-1 sequences from other species (Table S1). The phylogenetic tree (Fig. S2) clustered the inflammatory and apoptogenic caspases into two different clades. Within the inflammatory caspase clade, the fish caspases cluster into a single distinct branch with two subgroups: one for sea bass and sea bream caspase-1, and another one for the two caspy sequences (caspase-a and -b) from zebrafish.

### Sea bass caspase-1 is activated to p24/p10 and p20/p10 heterodimers in vitro

In order to characterize caspase-1 auto-processing and IL-1β activation in fish, a recombinant form of sea bass caspase-1 (DilaCASP1iso1) was produced.

**Figure 2:**
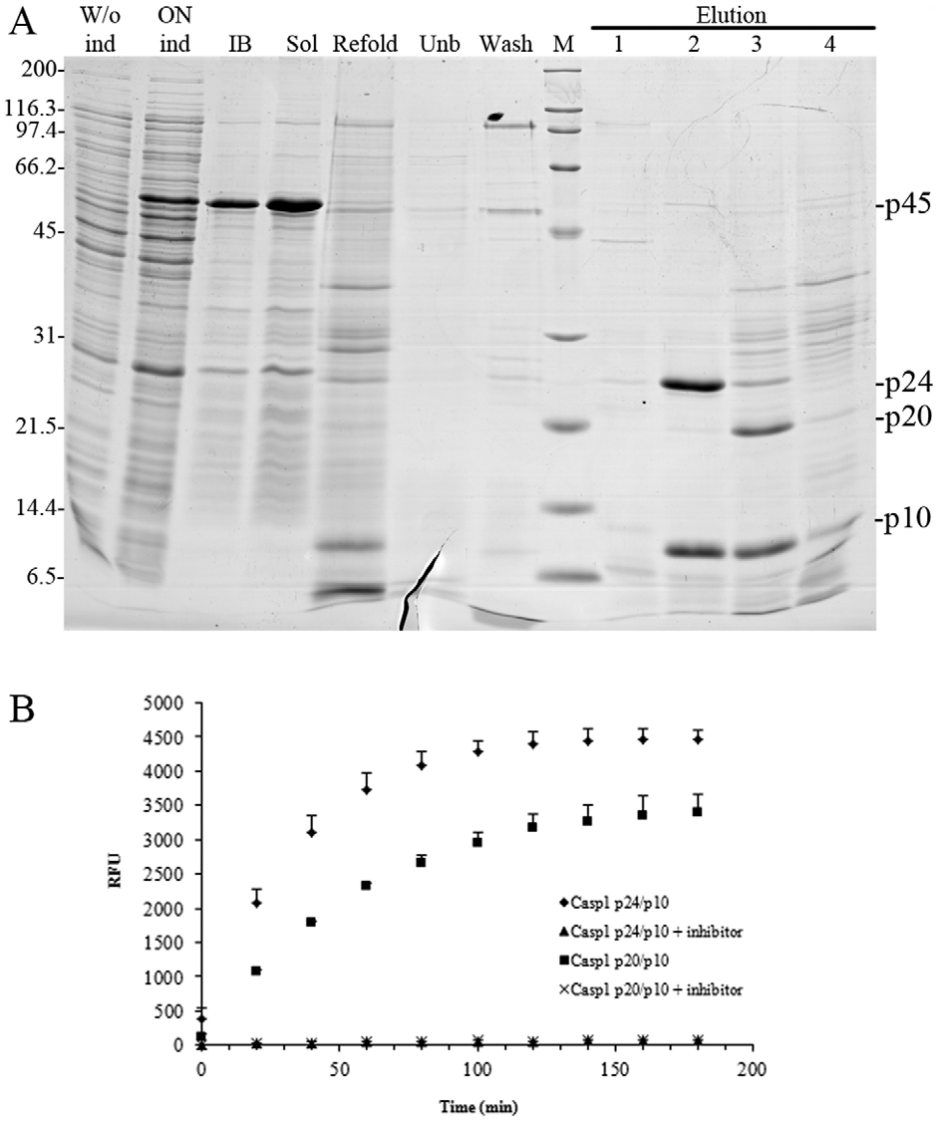
Recombinant sea bass caspase-1 displays enzymatic activity. (**A**) Production and purification of recombinant sea bass caspase-1. W/o ind: non induced; ON ind: overnight induction; IB: inclusion bodies' fraction; Sol: soluble fraction; Refold: overnight refolding; Unb, Wash and Elution: non-bound, washed and eluted (1–4) fractions, respectively, of Ni-NTA chromatography purified refolded caspase-1. The positions of the p45, p24, p20 and p10 fragments are indicated. Numbers on the left indicate the position of the molecular weight markers in kDa. (**B**) Enzymatic activity of recombinant p24/p10 and p20/p10 sea bass caspase-1 heterodimers. p24/p10 and p20/p10 were standardized for 1 μg of p10 and were incubated with a specific fluorogenic substrate for caspase-1 in the presence or absence of the caspase-1 inhibitor Ac-YVAD-CHO. Hydrolysis of the substrate was followed by fluorimetry and results were expressed as relative fluorescent units (RFU).

Solubilization and refolding of recombinant DilaCASP1iso1 from inclusion bodies originated three major polypeptides, two corresponding to putative large subunits (p24 and p20), and one corresponding to a putative small subunit (p10; Fig. 2A). N-terminal sequencing of the three polypeptides revealed that they were generated by proteolytic cleavage of sea bass caspase-1 after E^86^, D^108^ and D^304^ originating a p24, a p20, and a p10 polypeptide, respectively. Both p24/p10 and p20/p10 heterodimers (elutions #2 and #3, respectively, in Fig. 2A) displayed activity in a caspase-1 specific fluorimetric assay, which was abolished by the caspase-1 inhibitor Ac-YVAD-CHO (Fig. 2B). The more abundant p24/p10 heterodimer was selected for subsequent experiments.

**Figure 3:**
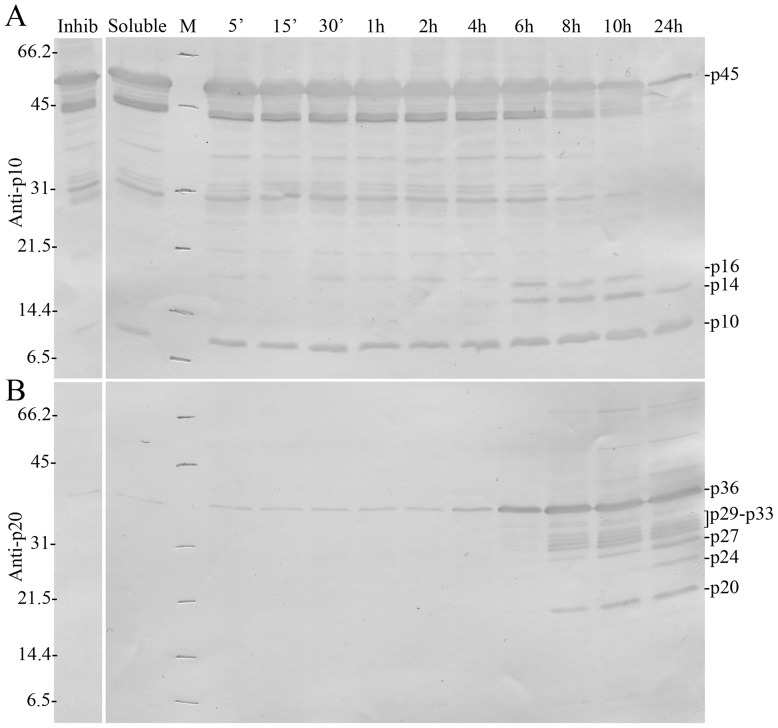
*In vitro* auto-processing of sea bass caspase-1. (**A**) Western blotting analysis of *in vitro* auto-processing of sea bass caspase-1 using a polyclonal antibody that detects all polypeptides containing the p10 domain. (**B**) Western blotting analysis of *in vitro* auto-processing of sea bass caspase-1 using a polyclonal antibody that detects only caspase-1 fragments that have been processed at the p20 cleavage site, i.e. that have released the linker plus the p10 domain. Soluble: solubilized DilaCASP1iso1. Inhib: sample incubated for 24 h in the presence of the specific caspase-1 inhibitor Ac-YVAD-CHO. The position of the different polypeptide fragments is indicated. Numbers on the left indicate the mass of the molecular weight (M) markers in kDa.

A kinetic analysis of the caspase-1 refolding process revealed a time-dependent decrease of the higher molecular weight forms and an increase of lower molecular weight forms (Fig. 3). The polypeptides detected in these experiments as well as the non-linear kinetics of processing, typical of an autocatalytic event, are remarkably similar to those described for the human protein [56], [57].

### DilaCASP1iso2 and DilaCASP1iso3 have little auto-processing activity but are processed by DilaCASP1iso1

**Figure 4:**
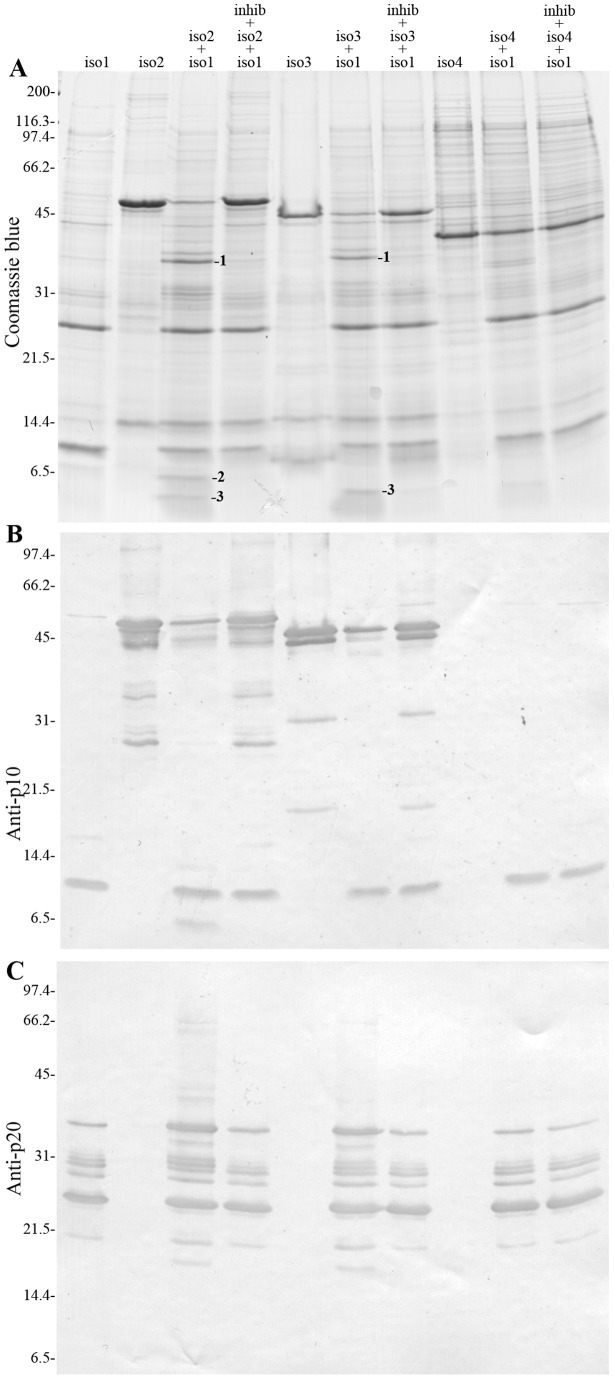
*In vitro* auto-processing and processing of sea bass caspase-1 isoforms 2, 3 and 4 by isoform 1. (**A**) Coomassie brilliant blue stained SDS-PAGE of sea bass DilaCASP1iso2 (iso2), DilaCASP1iso3 (iso3) or DilaCASP1iso4 (iso4) incubated alone or in combination with active (p24/p10) DilaCASP1iso1 (iso1), in the presence or absence of caspase-1 inhibitor Ac-YVAD-CHO (inhib). N-terminal sequencing revealed that the indicated fragments 1 and 2 correspond to p36 (GSSHHHHHHM^1^ADK) and p10 (T^305^LHFVH), respectively. Fragment 3 corresponds to an N-terminal fragment of p45 (GSSHHHHHHM^1^ADK). (**B**) Western blotting of the *in vitro* processing of caspase-1 isoforms as in A, using the anti-p10 polyclonal antibody that detects all polypeptides containing the p10 domain. (***C***) Western blotting of the *in vitro* processing of caspase-1 isoforms as in A, using the anti-p20 polyclonal antibody that detects only caspase-1 fragments that have been processed at the p20 cleavage site, i.e. that have released the linker plus the p10 domain. Numbers on the left indicate the mass of the molecular weight markers in kDa.

Considering that all spliced sea bass caspase-1 isoforms contain intact pro- and p20 domains (which include the active site), recombinant forms of sea bass caspase-1 isoforms 2, 3 and 4 were produced in order to test their auto-processing activity and/or processability by active DilaCASP1iso1 (Fig. 4). Although isoforms 2 and 3 showed auto-processing activity, which was totally absent in isoform 4 (Fig. 4), they do not generate active forms towards a specific substrate, as assessed by the fluorimetric assay. Moreover, isoforms 2 and 3, but not isoform 4, are readily processed by DilaCASP1iso1 (Fig. 4) with a fragment corresponding to the shorter p10 of isoform 2 being detected (Fig. 4A and B). For isoform 3, the shorter p10 fragment could not be detected probably because it is too small to be detected in these gels (Fig. 4A and B). The identity of the fragments was confirmed by N-terminal sequencing of p36 (GSSHHHHHHM^1^ADK; band 1 in Fig. 4A) and p10 (T^305^LHFVH; band 2 in Fig. 4A). Fragment 3 was a short fragment from the N-terminus (GSSHHHHHHM^1^ADK; band 3 in Fig. 4A). These results suggest that both isoforms 2 and 3 are processed by isoform 1, releasing the prodomain and the p10 plus linker.

### Evolutionary divergence of the caspase-1 cleavage site in proIL-1β

One of the main cellular substrates of caspase-1 is proIL-1β, although this has only been shown for mammals with no information available for non-mammalian species [39], [40], [41]. To evaluate the involvement of sea bass caspase-1 in proIL-1β processing, the enzyme was incubated either with *in vitro* translated sea bass IL-1β forms or recombinant sea bass proIL-1β.

**Figure 5:**
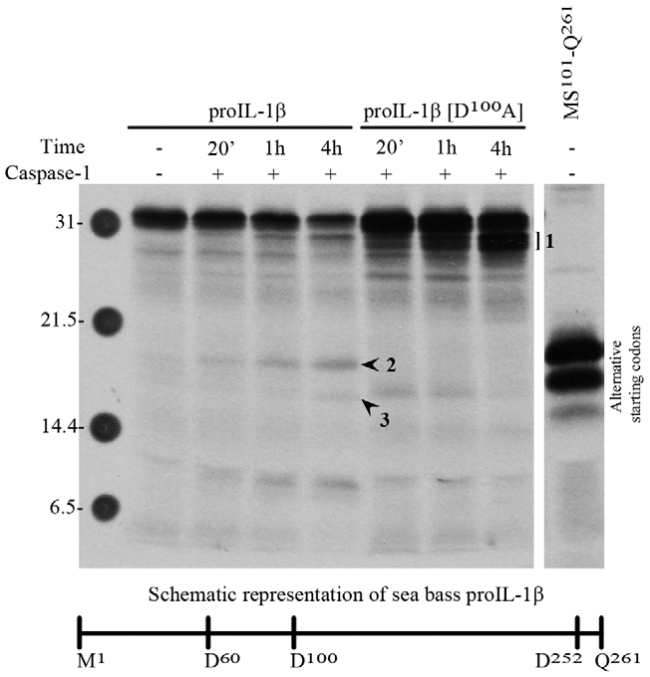
Cleavage of *in vitro* translated bass proIL-1β and proIL1β[D^100^A] by sea bass caspase-1. The same volume of *in vitro* synthesized proIL1β and proIL1β[D^100^A] were used and loaded on the gel. Fragments 1, 2 and 3 are labeled. Putative mature *in vitro* synthesized sea bass IL1β (MS^101^-Q^261^) has been loaded as control. Numbers on the left indicate the mass of the molecular weight markers in kDa. The double bands corresponding to the highest molecular weight form (fragment 1) suggested cleavage at the C-terminal end of proIL-1β. This interpretation was supported by N-terminal sequencing of fragment 1′ (Fig. S3B), which revealed that this fragment has the N-terminal sequence of proIL-1β (M^1^ESEMKC). Furthermore, mutation of D^252^ results in a proIL-1β protein that no longer yields fragment 1 upon incubation with caspase-1 (Fig S3C).

As shown in figure 5, *in vitro* synthesized sea bass proIL-1β was processed by sea bass active caspase-1 in a time-dependent manner giving rise to a band with an apparent molecular mass of 18 kDa that co-migrates with the putative mature sea bass IL-1β MS^101^-Q^261^. Mutation of D^100^ abolished the generation of the 18 kDa fragment, with accumulation of higher molecular mass species (Fig. 5), whereas mutating D^60^, a residue previously suggested as a possible caspase-1 cleavage site in fish [31], did not affect proIL-1β processing (Fig. S3A). This suggests that cleavage of sea bass proIL-1β by caspase-1 occurs C-terminal to D^100^, an aspartate residue absolutely conserved in all IL-1β sequences reported so far (Fig. S4).

An 18 kDa fragment was also generated from recombinant proIL-1β (Fig. S3B), together with a shorter fragment (fragment 3′) the intensity of which markedly increased with time. N-terminal sequencing of fragments 2′ and 3′ (Fig. S3B) revealed that they start at S^101^EKRSLVLVP, confirming that they originated from a cleavage after D^100^. Fragment 3′ was also processed after D^252^, as mutating D^252^ abolished its formation (Fig. S3C). Using *in vitro* translated proIL-1β it was confirmed that fragment 3′ appears upon long incubation periods (Fig. S3A, C). Furthermore, only few fish species present a slightly longer C-terminal region including potential cleavable aspartates (Fig. S4).

**Figure 6:**
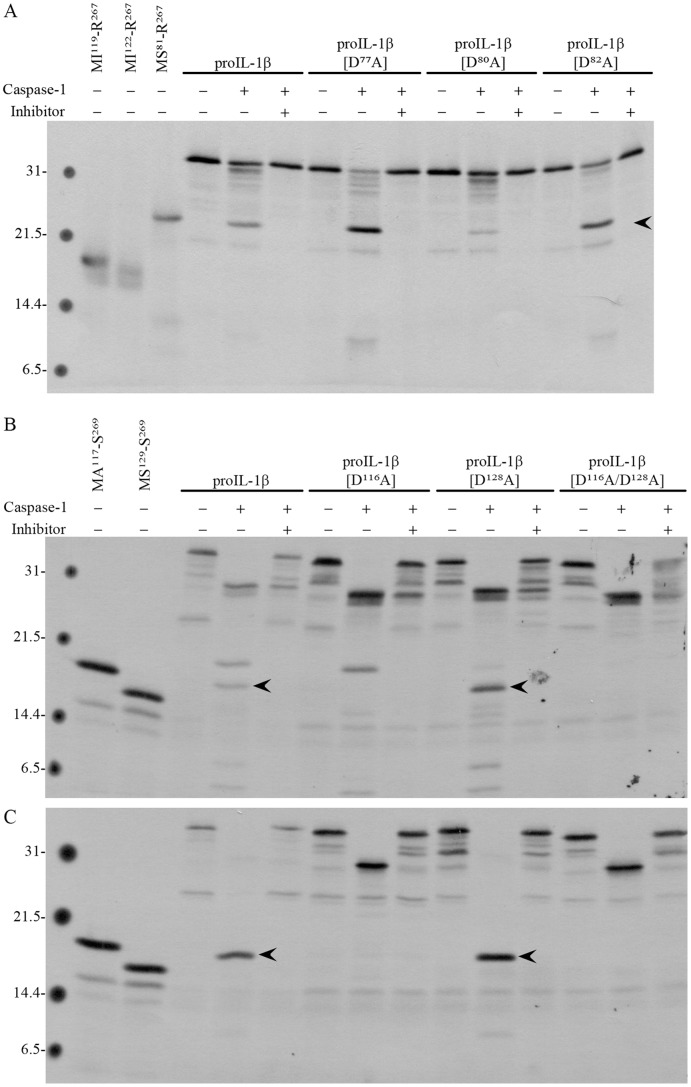
Processing of *in vitro* translated chicken and human proIL-1β by sea bass caspase-1. (**A**) Processing of *in vitro* translated chicken proIL-1β and mutants by sea bass caspase-1. The same volume of *in vitro* translated chicken proIL-1β, proIL1β[D^77^A], proIL1β[D^80^A] and proIL1β[D^82^A] were loaded on the gel. *In vitro* translated putative mature chicken IL-1β forms (MI^119^-R^267^, MI^122^-R^267^ and MS^81^-R^267^) were loaded as controls. (**B**) Processing of *in vitro* translated human proIL-1β and mutants by sea bass caspase-1. (**C**) Processing of *in vitro* translated human proIL-1β and mutants by human caspase-1. The same volume of *in vitro* tranlsated proIL-1β , proIL1β[D^116^A], proIL1β[D^128^A] and proIL1β[D^116^A/D^128^A] was loaded on the gel. *In vitro* translated mature human IL-1β (MA^117^-S^269^) and human IL-1β form (MS^129^-S^269^) starting at S^129^, homologue to sea bass S^101^, were loaded as controls. Mature forms are highlighted by arrow heads. Numbers on the left indicate the mass of the molecular weight markers in kDa.

As the caspase-1 cleavage site described for mammalian proIL-1β is only present in mammals (Fig. S4), it is tempting to speculate that the phylogenetically conserved aspartate identified as the caspase-1 cleavage site in sea bass proIL-1β is also the residue targeted by caspase-1 in the proIL-1β of other non-mammalian vertebrates. To test this possibility, the processing of *in vitro* translated avian pro-IL1β was investigated. Since avian caspase-1 is not available, sea bass or human caspase-1 was used. Both sea bass (Fig. 6A) and human (Fig. S5A) caspase-1 processed chicken proIL-1β into a mature IL-1β running in SDS-PAGE above the 21 kDa marker. A similar fragment was obtained when sea bass caspase-1 was incubated with duck, goose or turkey pro-IL1β (Fig. S5B). The apparent molecular mass of this fragment suggests that avian proIL-1β is cleaved at D^77^ or D^80^ or D^82^, which also align with other phylogenetically conserved aspartates (Fig. S4). Site-directed mutagenesis suggested that D^80^ is the preferential aspartate cleavage site, because it is the residue that upon mutation more drastically reduces the appearance of the putative mature form (Fig. 6A). Despite the concerns associated with the use of heterologous caspases, three observations suggest that they are cleaving the substrate specifically: (i) sea bass caspase-1 cleaved human proIL-1β at the specific mammalian caspase-1 cleavage site, originating the same mature form obtained by incubation with the human enzyme (Fig. 6B); (ii) despite the presence of several aspartates in the sea bass pro-IL1β, the human caspase-1 was unable to cleave sea bass pro-IL1β (Fig. S5C); (iii) a similar result was obtained when chicken proIL-1β was cleaved by caspase-1 from two phylogenetically distinct species.

#### In vivo-generated sea bass mature IL-1β is an 18 kDa polypeptide

**Figure 7:**
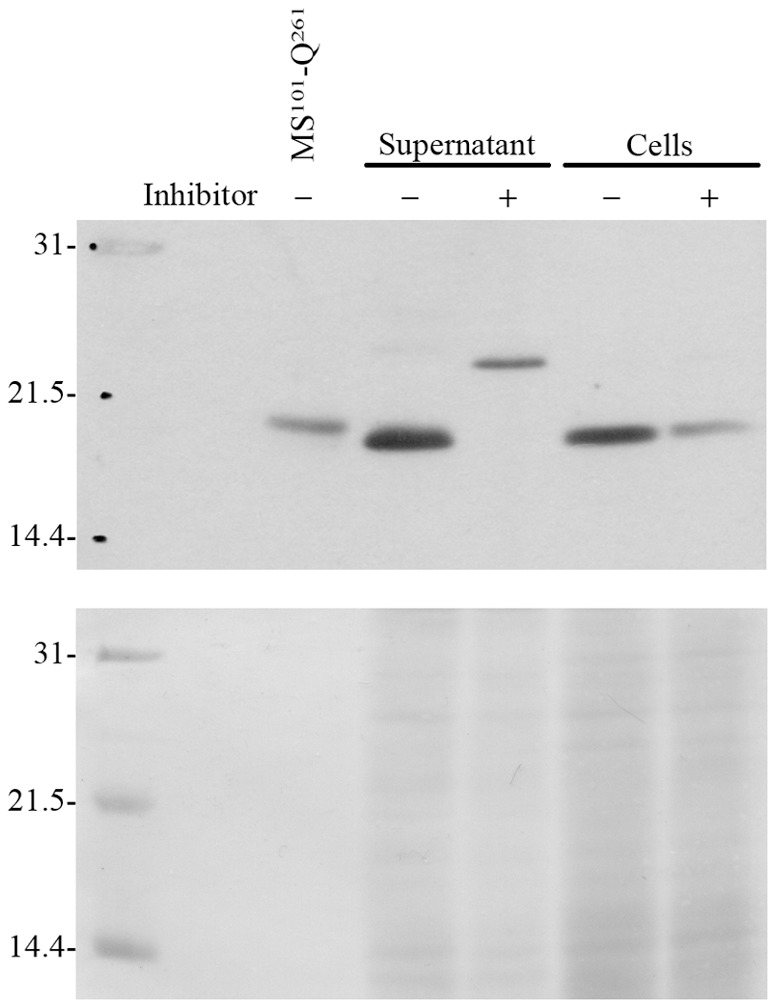
Sea bass IL-1β processing *in vivo*. (**A**) Western blotting using the anti-sea bass IL-1β polyclonal antibody. (**B**) Ponceau staining showing the protein loading. Inhibitor: specific caspase-1 inhibitor Ac-YVAD-CHO. Recombinant putative mature sea bass IL-1β (MS^101^-Q^261^) was loaded as control. Supernatant and Cells denote TCA precipitates of the supernatants and cell pellet derived from 1 ml of cell suspension. Numbers on the left indicate the mass of the molecular weight markers in kDa.

In order to obtain evidence supporting the *in vitro* cleavage results described above, peritoneal leukocytes harvested from stimulated sea bass were incubated in the presence or absence of a caspase-1 inhibitor. Western blotting analysis using an anti-sea bass IL-1β antibody, which recognizes proIL-1β and processed IL-1β fragments, showed a specific band with an apparent molecular mass of 18 kDa in culture supernatants and total extracts of cells incubated *ex vivo* without caspase-1 inhibitor (Fig. 7). Importantly, the 18 kDa protein co-migrates with a recombinant putative mature sea bass IL-1β starting after aspartate D^100^ (MS^101^-Q^261^). As there are no aspartates in sea bass proIL-1β sequence that could generate an 18 kDa polypeptide upon cleavage, other than D^100^, this suggests that *in vivo* sea bass proIL-1β is processed at D^100^, and does not suffers any additional cleavage at the C-terminal end (D^252^).

## Discussion

Caspase-1 primary structure has since long been known in humans [17], [18], [70] and mice [68], [55], but was only recently determined for two fish: sea bass (GenBank: DQ198376 and DQ198377) and sea bream [59]. Comparative analyses of the gene and of the primary structure of sea bass caspase-1 indicate that a strikingly conserved structure has been kept throughout evolution, suggesting that its function, specificity and processing mechanisms are highly conserved from at least teleost fish to mammals.

In humans, in addition to the caspase-1 isoform α, four mRNA isoforms (β, γ, δand ε) resulting from one or more alternative splicing events were described [71]. In the present work, besides sea bass caspase-1 isoform 1 (DilaCASP1iso1), three alternatively spliced mRNAs encoding DilaCASP1iso2, DilaCASP1iso3, and DilaCASP1iso4 were identified (Figs. 1 and S1B). Contrary to some of the human isoforms, where the prodomain (isoform γ) or both the prodomain and the region containing the active site (isoforms δ and ε) have been deleted [71], all sea bass isoforms contain the pro- and p20 domains, including the fully conserved active site QACRG sequence. The differences introduced by the alternative splicing events in sea bass caspase-1 isoforms are all located downstream from the active site, with DilaCASP1iso2 and DilaCASP1iso3 displaying a shorter p10 domain with a different amino acid sequence at the C-terminal end. DilaCASP1iso4 has a longer p20 domain but no p10 (Fig. S1B). The presence of alternative transcript variants of caspase-1 in sea bass and humans [72], [73] suggests that their existence has been conserved throughout evolution and therefore they are likely to play a functional role, as described for other caspase isoforms (see below).

Similarly to what has been described for human caspase-1 [17], [74], [56], [57], DilaCASP1iso1 is autocatalytically converted *in vitro* into two active heterodimeric complexes consisting of p10 paired either with p24 or p20 (Figs. 2A and 3). Strikingly, as for *Drosophila melanogaster* DRONC [75], sea bass caspase-1 p24 is generated by cleavage after a glutamate residue (P1 position) instead of the aspartate described in mammalian caspases as an absolute requirement for enzyme specificity [13], [76], [17]. Conversion of sea bass recombinant p45 to p20/p10 and p24/p10 heterodimers occurs in a time-dependent manner through a series of intermediates resembling the auto-processing of the human caspase-1 precursor, in which the first step is the cleavage of p10 and linker from the p45 form, followed by proteolytic conversion of the resulting p36 into smaller and more active p20 and p24 forms [56], [57]. Apparently, processing of procaspase-1 through several intermediates has been evolutionarily maintained at least from teleost fish to humans.

Sea bass caspase-1 isoforms 2 and 3 display some auto-processing activity, although in a much lesser degree than isoform 1. Of notice is that none of the fragments resulting from auto-processing of isoforms 2 and 3 correspond to p24, p20 or p10 (Fig. 4B and C), explaining why no enzymatic activity could be detected. However, contrary to the human isoforms [71], both sea bass isoforms 2 and 3 are processed into their p36, p20 and shorter p10 subunits by isoform 1. These data do not allow to conclude whether isoforms 2 and 3 participate in IL-1β processing or isoform 1 activation *in vivo*. On the other hand, being largely inactive, they may participate in a direct inhibitory mechanism of caspase-1, together with DilaCASP1iso4, by competing through CARD-CARD interactions for the inflammasome complex that activates caspase-1, as has been proposed for other CARD-containing proteins such as COP, INCA, ICEBERG and caspase-12 (reviewed in [77]). Alternative isoforms have been implicated in the inhibition of caspase-9 [78], [79], [80], and more recently also of caspase-3, where a short isoform (caspase-3S) antagonizes its apoptotic activity [81], thereby playing a physiological role in regulating cell death. Therefore, sea bass caspase-1 isoforms could also play a role in the regulation of the inflammatory response. Moreover, processing of isoforms 2 and 3 by isoform 1 released shorter p10 fragments, which could compete with normal p10 to generate inhibitory p20/short p10 heterodimers, as suggested for human ICEε [71].

In mammals, IL-1β is a pro-inflamatory cytokine produced as a 31 kDa inactive precursor [9], [10] that is processed by caspase-1 cleavage after D^116^ in humans and D^117^ in mice, yielding a C-terminal secreted active form of 17.5 kDa [14], [15], [11], [12], [16], [17], [18]. The primary structure of fish proIL-1β is similar to that of the mammalian cytokine [20], [21], [22], [23], [24], [25], [26], [27], [28], [29], [30]. However, the caspase-1 cleavage site present in all mammalian proIL-1β molecules is absent in the proIL-1β sequences reported so far for non-mammalian vertebrates (Fig. S4 and [39], [40], [41]), and the actual cleavage site has been greatly debated since the first non-mammalian IL-1β was sequenced [22]. Cleavage of *in vitro* translated and recombinant sea bass proIL-1β by caspase-1 suggests that mature sea bass IL-1β results from processing of its pro-form at D^100^ giving rise to an 18 kDa polypeptide, in accordance with the form detected *in vivo* (Fig. 7). Future studies on the activity of this polypeptide will clarify whether it corresponds to the biologically active IL-1β or to an inactive fragment.

As aspartate residues corresponding to sea bass D^100^ are present in all vertebrate IL-1β sequences (Fig. S4), it would be tempting to speculate that non-mammalian proIL-1β would be cleaved at this site and, thus, the mammalian IL-1β cleavage site would constitute the exception rather than the norm. Particularly in chicken, this position could be the likely activation site, rather than the alanine residue that was considered for the putative mature protein that was structurally characterized [36], as that constitutes an improbable caspase-1 cleavage site. However, the results presented here suggest that avian proIL-1βs may not be cleaved at the equivalent sea bass D^100^, but instead preferentially cleaved after D^80^ (Fig. 6 and S5B), present in another proIL-1β stretch where aspartates are phylogenetically well conserved (Fig. S4). In other fish species, fragments with different molecular mass have been proposed as putative active IL-1β [82], [37], [38], [31], [83], [31], and aspartate D^60^ in sea bream, equivalent to chicken D^80^, has even been suggested as a possible cleavage site, because processing at D^60^ would generate a fragment with a molecular mass consistent with the apparent size (22 kDa) of the IL-1β fragment detected by Western blotting in that species [31]. However, not only none of the reported studies included amino acid sequencing of the processed IL-1β fragments, but mutation of D^60^ did not affect at all sea bass IL-1β processing (Fig. S3A), in opposition to mutation of D^100^ where IL-1β processing to an 18 kDa form was abrogated (Fig. 6, S3A,C). To which extent processing of proIL-1β at this position is the norm among fish remains to be elucidated. The most obvious implication of sea bass proIL-1β processing at D^100^ is that the resulting mature form will be necessarily different from a structural viewpoint from the mammalian and chicken counterparts. In homology models of sea bass IL-1β produced using the available experimental structures (PDB entries 1IOB (human), 8I1B (mouse) or 2WRY (chicken)) as templates (not shown), residue D^100^ is located in a beta sheet-forming N-terminal segment. Therefore, structural conservation in this region would implicate the ablation of the N-terminal beta strand (which in the mammalian and chicken IL-1βs has an important structural function) and concomitant destabilization of the other C-terminal beta sheet-forming segments of the molecule upon cleavage of sea bass IL-1β at D^100^. Notably, in the mammalian proIL-1β caspase-1 cleavage site the P1′ position is almost always occupied by a small hydrophobic amino acid, which has been described as one of the preferential features of the peptide substrates of mammalian caspase-1 [13], [17]. On the contrary, most of the aspartates in the putative caspase-1 cleavage sites of non-mammalian vertebrates, sequence-wise to sea bass D^100^ or chicken D^80^, are not followed by a small hydrophobic amino acid. Also of notice is the fact that a small hydrophobic amino acid is present in the P1′ position after the phylogenetically conserved sea bass caspase-1 cleavage sites in monkeys (A^129^ in P1′), raising questions about the evolutionary driving force that resulted in the appearance of the mammalian cleavage site, as the mammalian aspartate at position P1 is only conserved in mammalian IL-1β sequences.

In conclusion, the present work shows that in a teleost fish, pro-caspase-1 auto-processing occurs through a series of intermediates, yielding active p24/p10 and p20/p10 heterodimers in a similar way to that described previously for human caspase-1. Moreover, the existence of alternative spliced variants of caspase-1 in sea bass is reported, suggesting that caspase-1 isoforms have been evolutionarily maintained and therefore likely play a regulatory role in the inflammatory response, as shown for isoforms of other caspases. Finally, it is shown that sea bass IL-1β is cleaved by caspase-1 at a phylogenetically conserved aspartic acid present in all known IL-1β sequences. However, in avian proIL-1βs another well phylogenetically conserved aspartate, only absent in humans, chimpanzees and zebrafish, may be apparently the cleavage site, suggesting that IL-1β processing may be class and/or even species specific.

## Supporting Information

Figure S1 Caspase-1 is encoded by a single gene in sea bass that encodes at least four different transcripts. (**A**) Southern blotting analysis. (**B**) Multiple sequence alignment of the predicted primary structures of sea bass caspase-1 isoforms and human capase-1α (HosaCASP1alpha). Sea bass caspase-1 comprises a pro-domain (Met^1^-Asp^108^), followed by a p20 domain (Gln^109^-Asp^278^), a short connecting peptide (Ser^279^-Asp^304^), and a p10 domain (Thr^305^-Leu^394^). The active-site pentapeptide QACRG is boxed. The aspartic acid cleavage sites are shaded in gray. Asterisks and “:” and “.” denote identity and chemical similarity between amino acids according to the default CLUSTAL W scoring matrix. The amino acid residues in the sea bass caspase-1 isoforms 2, 3 and 4 that differ from the isoform 1 are in bold. DilaCASP1iso1 (GenBank accession number: DQ198376) has 1663 bp including, as in human and mouse caspase-1 mRNA [18], [68], two polyadenylation signals within the 3′-UTR. The ORF encodes a 394 aa long protein that, as in the human and mouse sequences, has no hydrophobic signal sequence. Sequence analysis revealed that the sea bass protein contains both caspase family p20 and p10 domain profiles and a caspase family active site signature (K^257^PKIIIIQACRG). A prodomain containing a caspase recruitment domain (CARD), typical of pro-inflammatory caspases, is also present. DilaCASP1iso2 (GenBank accession number: HQ398873) results from an alternative splicing event involving the utilization of an alternative splicing site located within intron 7, which leads to the retention of 31 nucleotides from this intron (Fig. 1). This creates a putative caspase-1 isoform containing the complete pro- and p20 domains but having a shorter p10 domain (72 aa compared to 90 aa of the full length p10). DilaCASP1iso3 (GenBank accession numbers: HQ398874) is generated by an exon skipping event, in which exon 7 is lost (Fig. 1). This creates a putative caspase-1 isoform containing the complete pro- and p20 domains but having a much shorter p10 domain (25 aa compared to 90 aa of the full length p10). DilaCASP1iso4 (GenBank accession number: HQ398875) results from two alternative splicing events. One results in the retention of intron 5, generating a stop codon located 62 nucleotides downstream the beginning of this intron. The other is generated by an exon skipping event, in which exon 7 is spliced out of the transcript, as in DilaCASP1iso3 (Fig. 1). This creates a putative caspase-1 isoform containing a complete pro-domain; however, the splicing originates a shift in the reading frame that generates a different coding region after the active center. Although there is an aspartate residue within this alternative coding region, it is apparently not used as cleavage site, as this isoform is not auto-processed nor processed by active DilaCASP1iso1 (see below), therefore rendering a longer p20 domain (11 aa longer and 21 aa different) and no p10. (**C**) Expression analysis of caspase-1 isoforms in sea bass spleen.
(TIF)

Figure S2 Neighbour-joining tree (*MEGA* version 3.1) [63] with p-distance and complete deletion of gaps of inflammatory (caspase-1, −4, −5, −11 and −12) and apoptogenic (caspase-3 and −9) caspases. The amino acid sequences were aligned with CLUSTAL W [62] using the default parameters. The numbers in branching nodes denote the bootstrap percentages for 1000 replicates. The different branches are supported by high bootstrap values. GenBank accession numbers: for caspase-1 see Table S2; for caspase1/4: ABX79372 (*Canis lupus familiaris*); for caspase-3: ABC70996 (*Dicentrarchus labrax*), AAM43816 (*Takifugu rubripes*), BAB32409 (*Danio rerio*), CAC88866 (*Homo sapiens*), AAH81854 (*Rattus norvegicus*), AAH38825 (*Mus musculus*); for caspase-4: NP_001216 (*H. sapiens*), NP_788811 (*Bos taurus*), NP_446188 (*R. norvegicus*); for caspase-5: ABB58698 (*H. sapiens*), XP_001100375 (*Macaca mulatta*); for caspase-9: BAA87905 (*H. sapiens*), AAK26235 (*R. norvegicus*), AAH56447 (*M. musculus*), ABC70998 (*D. labrax*); the sequence of *Tetraodon nigroviridis* putative caspase-9 (CAG01765) was obtained by Blast search of the Genbank database with the sequence of sea bass caspase-9 as query; for caspase-11: CAA73531 (*M. musculus*), AAK38735 (*R. norvegicus*); for caspase-12: AAT91067 (*M. musculus*), EDL78548 (*R. norvegicus*), ABG21363 (*M. mulatta*), NP001070704 (*C. lupus familiaris*), ABX79369 (*Felis catus*).
(DOC)

Figure S3 Processing of i*n vitro* synthesized and recombinant sea bass proIL-1β by caspase-1. (**A**) Time-course processing of *in vitro* synthesized sea bass proIL-1β proIL1β[D^60^A] and proIL1β[D^100^A] by sea bass caspase-1, in the presence or absence of caspase-1 inhibitor Ac-YVAD-CHO, using caspase-1 buffer. *In vitro* synthesized putative mature sea bass IL-1β(MS^101^-Q^261^) was loaded as control. (**B**) Western blotting showing the time-course processing of recombinant sea bass proIL-1β by caspase-1 using caspase-1 buffer. proIL-1β[His] (proIL-1β) and DilaCASP1iso1 (Casp1) were loaded as controls. On the right, a schematic illustration of the fragments obtained, as concluded from N-terminal sequencing, is shown; capital letters denote amino acid residues and the superscript numbers the amino acid position within the sea bass IL-1β sequence. (**C**) Time-course processing of *in vitro* synthesized sea bass proIL-1β proIL1β[D^60^A] and proIL1β[D^100^A] using caspase-1 buffer. *In vitro* synthesized mature sea bass IL-1β (MS^101^-Q^261^) was loaded as control. (**D**) Processing of *in vitro* synthesized sea bass proIL-1β with or without caspase-1 buffer and with different amounts of caspase-1 (relative amount of caspase-1 added to the reaction). *In vitro* synthesized mature sea bass IL-1β (MS^101^-Q^261^) was loaded as control. ProIL-1β fragment 1, 2 and 3 are indicated in (C) and (D). Numbers on the left (right in D) indicate the mass of the molecular weight markers in kDa. The same volume of each *in vitro* synthesized proIL-1β forms were used and loaded on the gel.
(TIF)

Figure S4 Alignment of IL-1β amino acid sequences from vertebrates belonging to different Classes. The regions having aspartate cleavage sites are shown (aspartates are shaded black). The sequences were aligned with CLUSTAL W [62] using the default parameters. GenBank accession numbers are shown within brackets. Aspartate residues are blue color and bold type. The caspase-1 cleavage site in mammalian IL-1β sequences is shaded yellow. The conserved aspartic acids homologous to the caspase-1 cleavage site in sea bass IL-1β sequence are shaded green. The conserved aspartic acids homologous to the putative caspase-1 cleavage site in avian IL-1β sequence are shaded red. Mutated aspartate residue in the sea bass and chicken IL-1β sequences are shaded black.
(DOC)

Figure S5 Processing of *in vitro* translated sea bass and chicken proIL-1β by human and sea bass caspase-1, respectively. (**A**) Chicken proIL-1β is processed by human caspase-1. Putative mature chicken IL-1β forms (MS^81^R^267^, MI^119^R^267^ and MI^122^R^267^) were loaded as controls. (**B**) Processing of *in vitro* synthesized chicken, duck, goose and turkey proIL-1β by sea bass caspase-1. Putative mature chicken IL-1β forms (MS^81^R^267^, MI^119^R^267^ and MI^122^R^267^) were loaded as controls. (**C**) Sea bass proIL-1β is not processed by human caspase-1. Numbers on the left indicate the mass of the molecular weight markers in kDa. The same volume of *in vitro* synthesized proIL-1β forms for each species was used and loaded on the gel.
(TIF)

Table S1 Primer sequences and application.
(DOC)

Table S2 Amino acid sequence conservation for caspase-1 of different species.
(DOC)

## References

[1] Sims JE, Smith DE (2010) The IL-1 family: regulators of immunity. Nat Rev Immunol 10: 89–102. 20081871 10.1038/nri2691

[2] Netea MG, Simon A, van de Veerdonk F, Kullberg B-J, Van der Meer JWM, et al (2010) IL-1Î^2^ Processing in Host Defense: Beyond the Inflammasomes. PLoS Pathog 6: e1000661. 20195505 10.1371/journal.ppat.1000661 PMC2829053

[3] Eder C (2009) Mechanisms of interleukin-1[beta] release. Immunobiology 214: 543–553. 19250700 10.1016/j.imbio.2008.11.007

[4] Dinarello CA (2009) Immunological and Inflammatory Functions of the Interleukin-1 Family. Annual Review of Immunology 27: 519–550. 10.1146/annurev.immunol.021908.132612 19302047

[5] Burns K, Martinon F, Tschopp J (2003) New insights into the mechanism of IL-1beta maturation. Curr Opin Immunol 15: 26–30. 12495729 10.1016/s0952-7915(02)00017-1

[6] Dinarello CA (1998) Interleukin-1 beta, interleukin-18, and the interleukin-1 beta converting enzyme. Ann N Y Acad Sci 856: 1–11. 9917859 10.1111/j.1749-6632.1998.tb08307.x

[7] O'Neill LA (2008) The interleukin-1 receptor/Toll-like receptor superfamily: 10 years of progress. Immunol Rev 226: 10–18. 19161412 10.1111/j.1600-065X.2008.00701.x

[8] Wang D, Zhang S, Li L, Liu X, Mei K, et al (2010) Structural insights into the assembly and activation of IL-1[beta] with its receptors. Nat Immunol 11: 905–911. 20802483 10.1038/ni.1925

[9] March CJ, Mosley B, Larsen A, Cerretti DP, Braedt G, et al (1985) Cloning, sequence and expression of two distinct human interleukin-1 complementary DNAs. Nature 315: 641–647. 2989698 10.1038/315641a0

[10] Auron PE, Webb AC, Rosenwasser LJ, Mucci SF, Rich A, et al (1984) Nucleotide sequence of human monocyte interleukin 1 precursor cDNA. Proc Natl Acad Sci U S A 81: 7907–7911. 6083565 10.1073/pnas.81.24.7907 PMC392262

[11] Black RA, Kronheim SR, Sleath PR (1989) Activation of interleukin-1 beta by a co-induced protease. FEBS Lett 247: 386–390. 2653864 10.1016/0014-5793(89)81376-6

[12] Kostura MJ, Tocci MJ, Limjuco G, Chin J, Cameron P, et al (1989) Identification of a monocyte specific pre-interleukin 1 beta convertase activity. Proc Natl Acad Sci U S A 86: 5227–5231. 2787508 10.1073/pnas.86.14.5227 PMC297594

[13] Howard AD, Kostura MJ, Thornberry N, Ding GJ, Limjuco G, et al (1991) IL-1-converting enzyme requires aspartic acid residues for processing of the IL-1 beta precursor at two distinct sites and does not cleave 31-kDa IL-1 alpha. J Immunol 147: 2964–2969. 1919001

[14] Cameron P, Limjuco G, Rodkey J, Bennett C, Schmidt JA (1985) Amino acid sequence analysis of human interleukin 1 (IL-1). Evidence for biochemically distinct forms of IL-1. J Exp Med 162: 790–801. 3875682 10.1084/jem.162.3.790 PMC2187801

[15] Limjuco G, Galuska S, Chin J, Cameron P, Boger J, et al (1986) Antibodies of predetermined specificity to the major charged species of human interleukin 1. Proc Natl Acad Sci U S A 83: 3972–3976. 3520562 10.1073/pnas.83.11.3972 PMC323647

[16] Hogquist KA, Nett MA, Unanue ER, Chaplin DD (1991) Interleukin 1 is processed and released during apoptosis. Proc Natl Acad Sci U S A 88: 8485–8489. 1924307 10.1073/pnas.88.19.8485 PMC52533

[17] Thornberry NA, Bull HG, Calaycay JR, Chapman KT, Howard AD, et al (1992) A novel heterodimeric cysteine protease is required for interleukin-1 beta processing in monocytes. Nature 356: 768–774. 1574116 10.1038/356768a0

[18] Cerretti DP, Kozlosky CJ, Mosley B, Nelson N, Van Ness K, et al (1992) Molecular cloning of the interleukin-1 beta converting enzyme. Science 256: 97–100. 1373520 10.1126/science.1373520

[19] Fantuzzi G, Ku G, Harding MW, Livingston DJ, Sipe JD, et al (1997) Response to local inflammation of IL-1 beta-converting enzyme- deficient mice. J Immunol 158: 1818–1824. 9029121

[20] Seppola M, Larsen AN, Steiro K, Robertsen B, Jensen I (2008) Characterisation and expression analysis of the interleukin genes, IL-1[beta], IL-8 and IL-10, in Atlantic cod (Gadus morhua L.). Molecular Immunology 45: 887–897. 17875325 10.1016/j.molimm.2007.08.003

[21] Wang Y, Wang Q, Baoprasertkul P, Peatman E, Liu Z (2006) Genomic organization, gene duplication, and expression analysis of interleukin-1[beta] in channel catfish (Ictalurus punctatus). Molecular Immunology 43: 1653–1664. 16280165 10.1016/j.molimm.2005.09.024

[22] Zou J, Grabowski PS, Cunningham C, Secombes CJ (1999) MOLECULAR CLONING OF INTERLEUKIN 1[beta] FROM RAINBOW TROUT ONCORHYNCHUS MYKISS REVEALS NO EVIDENCE OF AN ICE CUT SITE. Cytokine 11: 552–560. 10433801 10.1006/cyto.1998.0470

[23] Scapigliati G, Buonocore F, Bird S, Zou J, Pelegrin P, et al (2001) Phylogeny of cytokines: molecular cloning and expression analysis of sea bass Dicentrarchus labrax interleukin-1beta. Fish Shellfish Immunol 11: 711–726. 11759041 10.1006/fsim.2001.0347

[24] Bird S, Wang T, Zou J, Cunningham C, Secombes CJ (2002) The first cytokine sequence within cartilaginous fish: IL-1 beta in the small spotted catshark (Scyliorhinus canicula). J Immunol 168: 3329–3340. 11907090 10.4049/jimmunol.168.7.3329

[25] Corripio-Miyar Y, Bird S, Tsamopoulos K, Secombes CJ (2007) Cloning and expression analysis of two pro-inflammatory cytokines, IL-1[beta] and IL-8, in haddock (Melanogrammus aeglefinus). Molecular Immunology 44: 1361–1373. 16831460 10.1016/j.molimm.2006.05.010

[26] Fujiki K, Shin DH, Nakao M, Yano T (2000) Molecular cloning and expression analysis of carp (Cyprinus carpio) interleukin-1 beta, high affinity immunoglobulin E Fc receptor gamma subunit and serum amyloid A. Fish Shellfish Immunol. 10: 229–242. 10.1006/fsim.1999.0253 10938736

[27] Jiang S, Zhang D, Li J, Liu Z (2008) Molecular characterization, recombinant expression and bioactivity analysis of the interleukin-1[beta] from the yellowfin sea bream, Acanthopagrus latus (Houttuyn). Fish & Shellfish Immunology 24: 323–336. 18201905 10.1016/j.fsi.2007.11.020

[28] Lee DS, Hong SH, Lee HJ, Jun LJ, Chung JK, et al (2006) Molecular cDNA cloning and analysis of the organization and expression of the IL-1beta gene in the Nile tilapia, Oreochromis niloticus. Comp Biochem Physiol A Mol Integr Physiol 143: 307–314. 16451827 10.1016/j.cbpa.2005.12.014

[29] Lu D-Q, Bei J-X, Feng L-N, Zhang Y, Liu X-C, et al (2008) Interleukin-1[beta] gene in orange-spotted grouper, Epinephelus coioides: Molecular cloning, expression, biological activities and signal transduction. Molecular Immunology 45: 857–867. 17920124 10.1016/j.molimm.2007.08.009

[30] Pelegrin P, Garcia-Castillo J, Mulero V, Meseguer J (2001) Interleukin-1beta isolated from a marine fish reveals up-regulated expression in macrophages following activation with lipopolysaccharide and lymphokines. Cytokine 16: 67–72. 11683587 10.1006/cyto.2001.0949

[31] Pelegrin P, Chaves-Pozo E, Mulero V, Meseguer J (2004) Production and mechanism of secretion of interleukin-1[beta] from the marine fish gilthead seabream. Developmental & Comparative Immunology 28: 229–237. 14642889 10.1016/j.dci.2003.08.002

[32] Hong S, Zou J, Crampe M, Peddie S, Scapigliati G, et al (2001) The production and bioactivity of rainbow trout (Oncorhynchus mykiss) recombinant IL-1[beta]. Veterinary Immunology and Immunopathology 81: 1–14. 11498242 10.1016/s0165-2427(01)00328-2

[33] Buonocore F, Mazzini M, Forlenza M, Randelli E, Secombes CJ, et al (2004) Expression in Escherchia coli and purification of sea bass (Dicentrarchus labrax) interleukin 1beta, a possible immunoadjuvant in aquaculture. Mar Biotechnol (NY) 6: 53–59. 14612985 10.1007/s10126-003-0011-y

[34] Weining KC, Sick C, Kaspers B, Staeheli P (1998) A chicken homolog of mammalian interleukin-1 beta: cDNA cloning and purification of active recombinant protein. Eur J Biochem 258: 994–1000. 9990317 10.1046/j.1432-1327.1998.2580994.x

[35] Wu YF, Liu HJ, Chiou SH, Lee LH (2007) Sequence and phylogenetic analysis of interleukin (IL)-1beta-encoding genes of five avian species and structural and functional homology among these IL-1beta proteins. Vet Immunol Immunopathol 116: 37–46. 17275099 10.1016/j.vetimm.2006.12.010

[36] Cheng CS, Chen WT, Lee LH, Chen YW, Chang SY, et al (2011) Structural and functional comparison of cytokine interleukin-1 beta from chicken and human. Mol Immunol 48: 947–955. 21288573 10.1016/j.molimm.2011.01.002

[37] Hong S, Zou J, Collet B, Bols NC, Secombes CJ (2004) Analysis and characterisation of IL-1[beta] processing in rainbow trout, Oncorhynchus mykiss. Fish & Shellfish Immunology 16: 453–459. 15123312 10.1016/j.fsi.2003.08.002

[38] Mathew JA, Guo YX, Goh KP, Chan J, Verburg-van Kemenade BML, et al (2002) Characterisation of a monoclonal antibody to carp IL-1β and the development of a sensitive capture ELISA. Fish & Shellfish Immunology 13: 85–95. 12400859 10.1006/fsim.2001.0383

[39] Bird S, Zou J, Wang T, Munday B, Cunningham C, et al (2002) Evolution of interleukin-1beta. Cytokine Growth Factor Rev 13: 483–502. 12401481 10.1016/s1359-6101(02)00028-x

[40] Sakamaki K, Satou Y (2009) Caspases: evolutionary aspects of their functions in vertebrates. Journal of Fish Biology 74: 727–753. 20735596 10.1111/j.1095-8649.2009.02184.x PMC2779465

[41] Secombes CJ, Wang T, Bird S (2011) The interleukins of fish. Developmental & Comparative Immunology 35: 1336–1345. 21605591 10.1016/j.dci.2011.05.001

[42] Scott AM, Saleh M (2007) The inflammatory caspases: guardians against infections and sepsis. Cell Death Differ 14: 23–31. 16977333 10.1038/sj.cdd.4402026

[43] Ayala JM, Yamin TT, Egger LA, Chin J, Kostura MJ, et al (1994) IL-1 beta-converting enzyme is present in monocytic cells as an inactive 45-kDa precursor. J Immunol 153: 2592–2599. 8077669

[44] Strowig T, Henao-Mejia J, Elinav E, Flavell R (2012) Inflammasomes in health and disease. Nature 481: 278–286. 22258606 10.1038/nature10759

[45] Davis BK, Wen H, Ting JPY (2011) The Inflammasome NLRs in Immunity, Inflammation, and Associated Diseases. Annual Review of Immunology 29: 707–735. 10.1146/annurev-immunol-031210-101405 PMC4067317 21219188

[46] Latz E (2010) The inflammasomes: mechanisms of activation and function. Current Opinion in Immunology 22: 28–33. 20060699 10.1016/j.coi.2009.12.004 PMC2844336

[47] Pedra JHF, Cassel SL, Sutterwala FS (2009) Sensing pathogens and danger signals by the inflammasome. Current Opinion in Immunology 21: 10–16. 19223160 10.1016/j.coi.2009.01.006 PMC2701640

[48] Mohamed Lamkanfi VMD (2009) Inflammasomes: guardians of cytosolic sanctity. Immunological Reviews 227: 95–105. 19120479 10.1111/j.1600-065X.2008.00730.x

[49] McIntire CR, Yeretssian G, Saleh M (2009) Inflammasomes in infection and inflammation. Apoptosis 14: 522–535. 19156527 10.1007/s10495-009-0312-3

[50] Martinon F, Mayor A, Tschopp Jr (2009) The Inflammasomes: Guardians of the Body. Annual Review of Immunology 27: 229–265. 10.1146/annurev.immunol.021908.132715 19302040

[51] Franchi L, Eigenbrod T, Munoz-Planillo R, Nunez G (2009) The inflammasome: a caspase-1-activation platform that regulates immune responses and disease pathogenesis. Nat Immunol 10: 241–247. 19221555 10.1038/ni.1703 PMC2820724

[52] Brennan MA, Cookson BT (2000) *Salmonella* induces macrophage death by caspase-1-dependent necrosis. Molecular Microbiology 38: 31–40. 11029688 10.1046/j.1365-2958.2000.02103.x

[53] Bergsbaken T, Fink SL, Cookson BT (2009) Pyroptosis: host cell death and inflammation. Nat Rev Micro 7: 99–109. 10.1038/nrmicro2070 PMC2910423 19148178

[54] Miao EA, Rajan JV, Aderem A (2011) Caspase-1-induced pyroptotic cell death. Immunological Reviews 243: 206–214. 21884178 10.1111/j.1600-065X.2011.01044.x PMC3609431

[55] Molineaux S, Casano F, Rolando A, Peterson E, Limjuco G, et al (1993) Interleukin 1{beta} (IL-1{beta}) Processing in Murine Macrophages Requires a Structurally Conserved Homologue of Human IL-1{beta} Converting Enzyme. PNAS 90: 1809–1813. 8446594 10.1073/pnas.90.5.1809 PMC45969

[56] Ramage P, Cheneval D, Chvei M, Graff P, Hemmig R, et al (1995) Expression, refolding, and autocatalytic proteolytic processing of the interleukin-1 beta-converting enzyme precursor. J Biol Chem 270: 9378–9383. 7721861 10.1074/jbc.270.16.9378

[57] Yamin TT, Ayala JM, Miller DK (1996) Activation of the native 45-kDa precursor form of interleukin-1-converting enzyme. J Biol Chem 271: 13273–13282. 8662843 10.1074/jbc.271.22.13273

[58] Masumoto J, Zhou W, Chen FF, Su F, Kuwada JY, et al (2003) Caspy, a Zebrafish Caspase, Activated by ASC Oligomerization Is Required for Pharyngeal Arch Development. J Biol Chem 278: 4268–4276. 12464617 10.1074/jbc.M203944200

[59] Lopez-Castejon G, Sepulcre MP, Mulero I, Pelegrin P, Meseguer J, et al (2008) Molecular and functional characterization of gilthead seabream Sparus aurata caspase-1: The first identification of an inflammatory caspase in fish. Molecular Immunology 45: 49–57. 17610954 10.1016/j.molimm.2007.05.015

[60] Stet RJ, van Erp SH, Hermsen T, Sultmann HA, Egberts E (1993) Polymorphism and estimation of the number of MhcCyca class I and class II genes in laboratory strains of the common carp (Cyprinus carpio L.). Dev Comp Immunol 17: 141–156. 8099021 10.1016/0145-305x(93)90024-k

[61] Ausubel FM, Brent R, Kingston RE, Moore DD, Seidman JG, et al. (1999) Current Protocols in Molecular Biology. New York: John Wiley & Sons, Inc.

[62] Thompson JD, Higgins DG, Gibson TJ (1994) CLUSTAL W: improving the sensitivity of progressive multiple sequence alignment through sequence weighting, position-specific gap penalties and weight matrix choice. Nucleic Acids Res 22: 4673–4680. 7984417 10.1093/nar/22.22.4673 PMC308517

[63] Kumar S, Tamura K, Nei M (2004) MEGA3: Integrated software for Molecular Evolutionary Genetics Analysis and sequence alignment. Brief Bioinform 5: 150–163. 15260895 10.1093/bib/5.2.150

[64] Needleman SB, Wunsch CD (1970) A general method applicable to the search for similarities in the amino acid sequence of two proteins. J Mol Biol 48: 443–453. 5420325 10.1016/0022-2836(70)90057-4

[65] do Vale A, Silva MT, dos Santos NMS, Nascimento DS, Reis-Rodrigues P, et al (2005) AIP56, a novel plasmid-encoded virulence factor of Photobacterium damselae subsp. piscicida with apoptogenic activity against sea bass macrophages and neutrophils. Molecular Microbiology 58: 1025–1038. 16262788 10.1111/j.1365-2958.2005.04893.x

[66] Afonso A, Ellis AE, Silva MT (1997) The leucocyte population of the unstimulated peritoneal cavity of rainbow trout (Oncorhynchus mykiss). Fish & Shellfish Immunology 7: 335–348.

[67] Do Vale A, Afonso A, Silva MT (2002) The professional phagocytes of sea bass (Dicentrarchus labrax L.): cytochemical characterisation of neutrophils and macrophages in the normal and inflamed peritoneal cavity. Fish & Shellfish Immunology 13: 183–198. 12365730 10.1006/fsim.2001.0394

[68] Nett MA, Cerretti DP, Berson DR, Seavitt J, Gilbert DJ, et al (1992) Molecular cloning of the murine IL-1 beta converting enzyme cDNA. J Immunol 149: 3254–3259. 1431103

[69] Casano FJ, Rolando AM, Mudgett JS, Molineaux SM (1994) The structure and complete nucleotide sequence of the murine gene encoding interleukin-1 beta converting enzyme (ICE). Genomics 20: 474–481. 8034321 10.1006/geno.1994.1203

[70] Cerretti DP, Hollingsworth LT, Kozlosky CJ, Valentine MB, Shapiro DN, et al (1994) Molecular characterization of the gene for human interleukin-1 beta converting enzyme (IL1BC). Genomics 20: 468–473. 8034320 10.1006/geno.1994.1202

[71] Alnemri ES, Fernandes-Alnemri T, Litwack G (1995) Cloning and Expression of Four Novel Isoforms of Human Interleukin-1beta Converting Enzyme with Different Apoptotic Activities. J Biol Chem 270: 4312–4317. 7876192 10.1074/jbc.270.9.4312

[72] Chen M, Manley JL (2009) Mechanisms of alternative splicing regulation: insights from molecular and genomics approaches. Nat Rev Mol Cell Biol 10: 741–754. 19773805 10.1038/nrm2777 PMC2958924

[73] Keren H, Lev-Maor G, Ast G (2010) Alternative splicing and evolution: diversification, exon definition and function. Nat Rev Genet 11: 345–355. 20376054 10.1038/nrg2776

[74] Miller DK, Ayala JM, Egger LA, Raju SM, Yamin TT, et al (1993) Purification and characterization of active human interleukin-1 beta-converting enzyme from THP.1 monocytic cells. J Biol Chem 268: 18062–18069. 8349684

[75] Snipas SJ, Drag M, Stennicke HR, Salvesen GS (2008) Activation mechanism and substrate specificity of the Drosophila initiator caspase DRONC. Cell Death Differ 15: 938–945. 18309328 10.1038/cdd.2008.23

[76] Sleath PR, Hendrickson RC, Kronheim SR, March CJ, Black RA (1990) Substrate specificity of the protease that processes human interleukin-1 beta. J Biol Chem 265: 14526–14528. 2201686

[77] Guarda G, So A (2010) Regulation of inflammasome activity. Immunology 130: 329–336. 20465574 10.1111/j.1365-2567.2010.03283.x PMC2913212

[78] Angelastro JM, Moon NY, Liu DX, Yang AS, Greene LA, et al (2001) Characterization of a novel isoform of caspase-9 that inhibits apoptosis. J Biol Chem 276: 12190–12200. 11278518 10.1074/jbc.M009523200

[79] Seol D-W, Billiar TR (1999) A Caspase-9 Variant Missing the Catalytic Site Is an Endogenous Inhibitor of Apoptosis. J Biol Chem 274: 2072–2076. 9890966 10.1074/jbc.274.4.2072

[80] Srinivasula SM, Ahmad M, Guo Y, Zhan Y, Lazebnik Y, et al (1999) Identification of an Endogenous Dominant-Negative Short Isoform of Caspase-9 That Can Regulate Apoptosis. Cancer Res 59: 999–1002. 10070954

[81] Végran F, Boidot R, Solary E, Lizard-Nacol S (2011) A Short Caspase-3 Isoform Inhibits Chemotherapy-Induced Apoptosis by Blocking Apoptosome Assembly. PLoS ONE 6: e29058. 22216167 10.1371/journal.pone.0029058 PMC3245238

[82] Ellsaesser CF, Clem LW (1994) Functionally distinct high and low molecular weight species of channel catfish and mouse IL-1. Cytokine 6: 10–20. 8003627 10.1016/1043-4666(94)90002-7

[83] Verburg-van Kemenade BM, Weyts FA, Debets R, Flik G (1995) Carp macrophages and neutrophilic granulocytes secrete an interleukin-1-like factor. Dev Comp Immunol 19: 59–70. 7615138 10.1016/0145-305x(94)00047-j

