# Supplementary figures and images for: Caspase-1 and IL-1β Processing in a Teleost Fish
Source: PLoS One. 2012 Nov 30;7(11):e50450. doi: 10.1371/journal.pone.0050450 (PMC3511578; doi:10.1371/journal.pone.0050450)

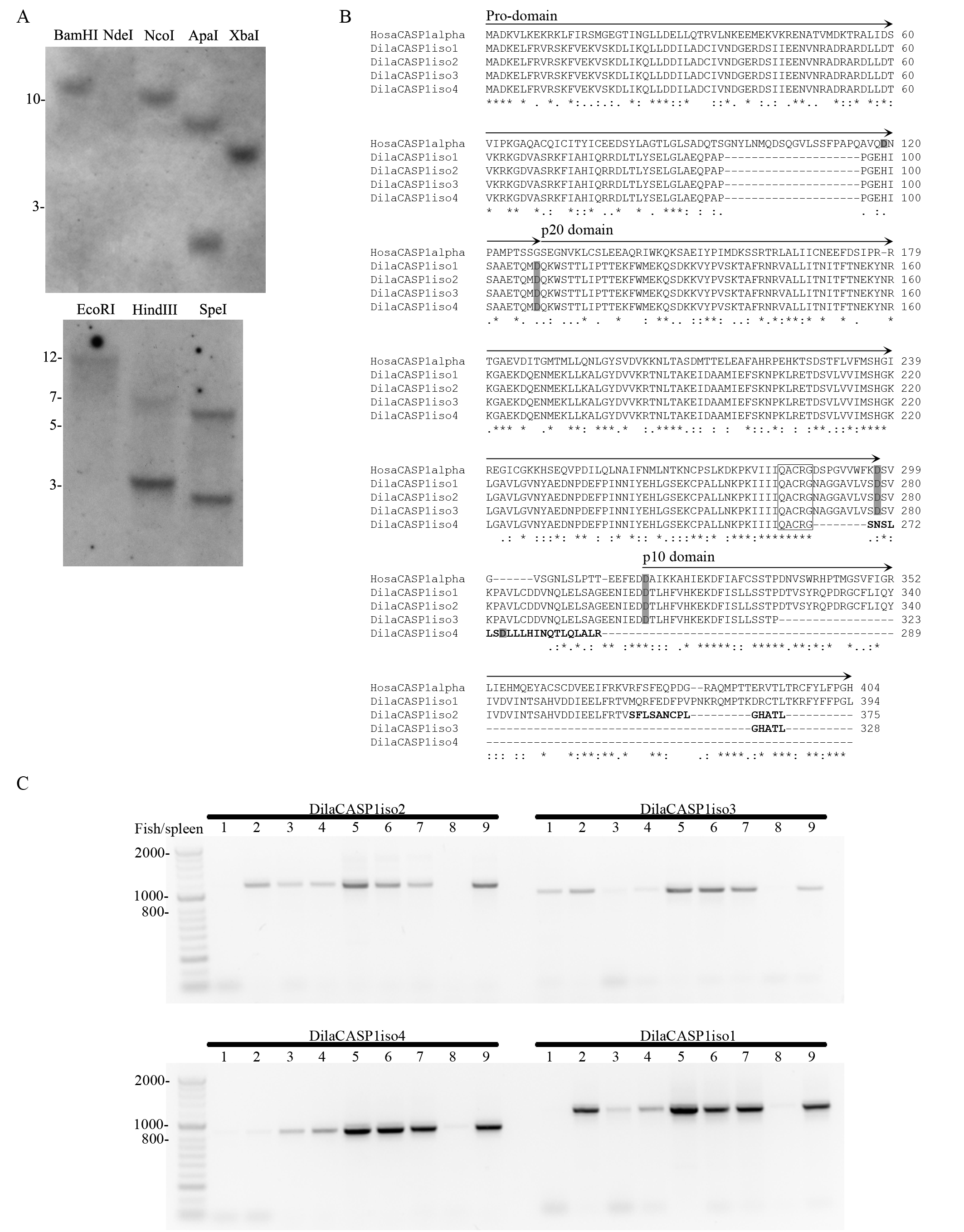

Supplement: Figure S1 — Caspase-1 is encoded by a single gene in sea bass that encodes at least four different transcripts. (A) Southern blotting analysis. (B) Multiple sequence alignment of the predicted primary structures of sea bass caspase-1 isoforms and human capase-1α (HosaCASP1alpha). Sea bass caspase-1 comprises a pro-domain (Met1-Asp108), followed by a p20 domain (Gln109-Asp278), a short connecting peptide (Ser279-Asp304), and a p10 domain (Thr305-Leu394). The active-site pentapeptide QACRG is boxed. The aspartic acid cleavage sites are shaded in gray. Asterisks and “:” and “.” denote identity and chemical similarity between amino acids according to the default CLUSTAL W scoring matrix. The amino acid residues in the sea bass caspase-1 isoforms 2, 3 and 4 that differ from the isoform 1 are in bold. DilaCASP1iso1 (GenBank accession number: DQ198376) has 1663 bp including, as in human and mouse caspase-1 mRNA [18], [68], two polyadenylation signals within the 3′-UTR. The ORF encodes a 394 aa long protein that, as in the human and mouse sequences, has no hydrophobic signal sequence. Sequence analysis revealed that the sea bass protein contains both caspase family p20 and p10 domain profiles and a caspase family active site signature (K257PKIIIIQACRG). A prodomain containing a caspase recruitment domain (CARD), typical of pro-inflammatory caspases, is also present. DilaCASP1iso2 (GenBank accession number: HQ398873) results from an alternative splicing event involving the utilization of an alternative splicing site located within intron 7, which leads to the retention of 31 nucleotides from this intron (Fig. 1). This creates a putative caspase-1 isoform containing the complete pro- and p20 domains but having a shorter p10 domain (72 aa compared to 90 aa of the full length p10). DilaCASP1iso3 (GenBank accession numbers: HQ398874) is generated by an exon skipping event, in which exon 7 is lost (Fig. 1). This creates a putative caspase-1 isoform containing the complete pro- and p20 domain [file pone.0050450.s001.tif]

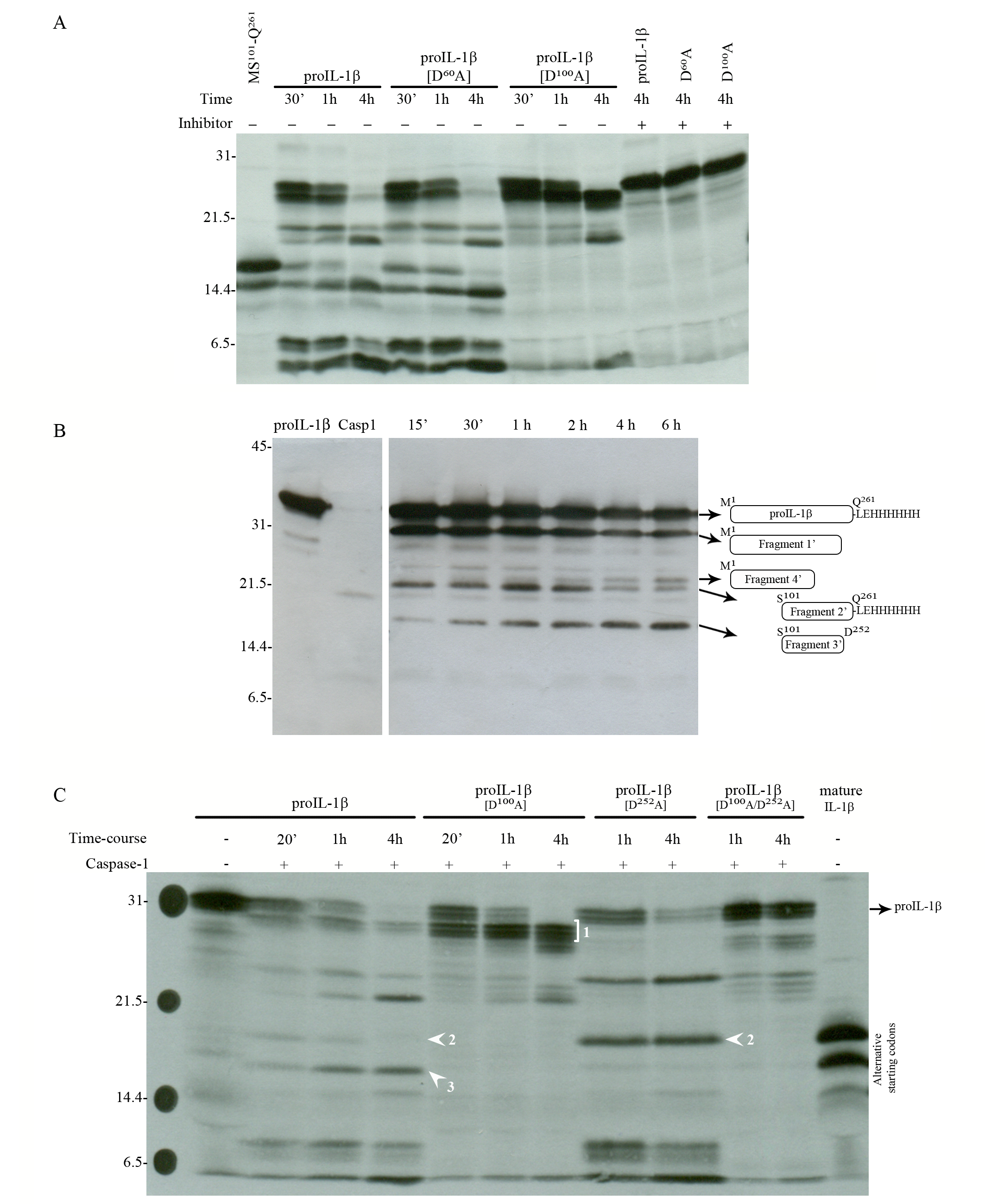

Supplement: Figure S3 — Processing of in vitro synthesized and recombinant sea bass proIL-1β by caspase-1. (A) Time-course processing of in vitro synthesized sea bass proIL-1β proIL1β[D60A] and proIL1β[D100A] by sea bass caspase-1, in the presence or absence of caspase-1 inhibitor Ac-YVAD-CHO, using caspase-1 buffer. In vitro synthesized putative mature sea bass IL-1β(MS101-Q261) was loaded as control. (B) Western blotting showing the time-course processing of recombinant sea bass proIL-1β by caspase-1 using caspase-1 buffer. proIL-1β[His] (proIL-1β) and DilaCASP1iso1 (Casp1) were loaded as controls. On the right, a schematic illustration of the fragments obtained, as concluded from N-terminal sequencing, is shown; capital letters denote amino acid residues and the superscript numbers the amino acid position within the sea bass IL-1β sequence. (C) Time-course processing of in vitro synthesized sea bass proIL-1β proIL1β[D60A] and proIL1β[D100A] using caspase-1 buffer. In vitro synthesized mature sea bass IL-1β (MS101-Q261) was loaded as control. (D) Processing of in vitro synthesized sea bass proIL-1β with or without caspase-1 buffer and with different amounts of caspase-1 (relative amount of caspase-1 added to the reaction). In vitro synthesized mature sea bass IL-1β (MS101-Q261) was loaded as control. ProIL-1β fragment 1, 2 and 3 are indicated in (C) and (D). Numbers on the left (right in D) indicate the mass of the molecular weight markers in kDa. The same volume of each in vitro synthesized proIL-1β forms were used and loaded on the gel. (TIF) [file pone.0050450.s003.tif]

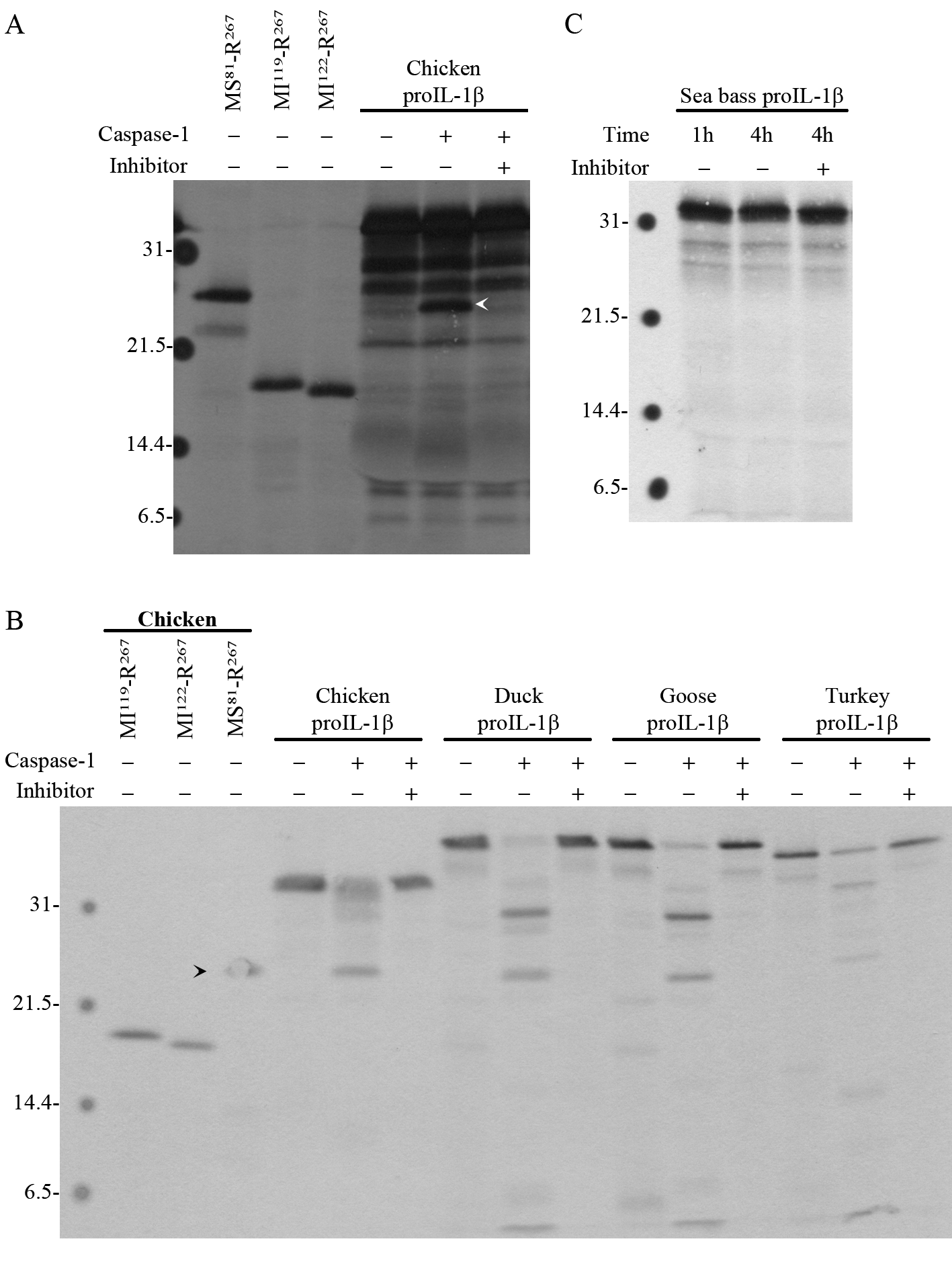

Supplement: Figure S5 — Processing of in vitro translated sea bass and chicken proIL-1β by human and sea bass caspase-1, respectively. (A) Chicken proIL-1β is processed by human caspase-1. Putative mature chicken IL-1β forms (MS81R267, MI119R267 and MI122R267) were loaded as controls. (B) Processing of in vitro synthesized chicken, duck, goose and turkey proIL-1β by sea bass caspase-1. Putative mature chicken IL-1β forms (MS81R267, MI119R267 and MI122R267) were loaded as controls. (C) Sea bass proIL-1β is not processed by human caspase-1. Numbers on the left indicate the mass of the molecular weight markers in kDa. The same volume of in vitro synthesized proIL-1β forms for each species was used and loaded on the gel. (TIF) [file pone.0050450.s005.tif]
