# Supplementary material for: Caspase-1 and IL-1β Processing in a Teleost Fish
Source: PLoS One. 2012 Nov 30;7(11):e50450. doi: 10.1371/journal.pone.0050450 (PMC3511578; doi:10.1371/journal.pone.0050450)
Supplement: Figure S2 — Neighbour-joining tree (MEGA version 3.1) [63] with p-distance and complete deletion of gaps of inflammatory (caspase-1, −4, −5, −11 and −12) and apoptogenic (caspase-3 and −9) caspases. The amino acid sequences were aligned with CLUSTAL W [62] using the default parameters. The numbers in branching nodes denote the bootstrap percentages for 1000 replicates. The different branches are supported by high bootstrap values. GenBank accession numbers: for caspase-1 see Table S2; for caspase1/4: ABX79372 (Canis lupus familiaris); for caspase-3: ABC70996 (Dicentrarchus labrax), AAM43816 (Takifugu rubripes), BAB32409 (Danio rerio), CAC88866 (Homo sapiens), AAH81854 (Rattus norvegicus), AAH38825 (Mus musculus); for caspase-4: NP_001216 (H. sapiens), NP_788811 (Bos taurus), NP_446188 (R. norvegicus); for caspase-5: ABB58698 (H. sapiens), XP_001100375 (Macaca mulatta); for caspase-9: BAA87905 (H. sapiens), AAK26235 (R. norvegicus), AAH56447 (M. musculus), ABC70998 (D. labrax); the sequence of Tetraodon nigroviridis putative caspase-9 (CAG01765) was obtained by Blast search of the Genbank database with the sequence of sea bass caspase-9 as query; for caspase-11: CAA73531 (M. musculus), AAK38735 (R. norvegicus); for caspase-12: AAT91067 (M. musculus), EDL78548 (R. norvegicus), ABG21363 (M. mulatta), NP001070704 (C. lupus familiaris), ABX79369 (Felis catus). (DOC) [file pone.0050450.s002.doc]

**INFLAMMATORY CASPASES**

*Homo sapiens* caspase-4 alpha

*Bos taurus* caspase-4

*Homo sapiens* caspase-5a

*Macaca mulatta* caspase-5

*Felis catus* caspase-1

*Canis lupus familiaris* caspase-1/4

*Mus musculus* caspase-12

*Rattus norvegicus* caspase-12

*Macaca mulatta* caspase-12

*Canis lupus familiaris* caspase-12

*Felis catus* caspase-12a

*Mus musculus* caspase-11

*Rattus norvegicus* caspase-4

*Rattus norvegicus* caspase-11

*Homo sapiens* caspase-1

*Rattus norvegicus* caspase-1

*Mus musculus* caspase-1

*Gallus gallus* caspase-1

*Xenopus laevis* caspase-1

***Dicentrarchus labrax* caspase-1**

*Sparus aurata* caspase-1

*Danio rerio* caspase-a

*Danio rerio* caspase-b

*Mus musculus* caspase-9

*Rattus norvegicus* caspase-9

*Homo sapiens* caspase-9

*Dicentrarchus labrax* caspase-9

*Tetraodon nigroviridis* putative caspase-9

*Dicentrarchus labrax* caspase-3

*Takifugu rubripes* caspase-3

*Danio rerio* caspase-3

*Homo sapiens* caspase-3

*Rattus norvegicus* caspase-3

*Mus musculus* caspase-3

100

100

100

96

100

100

93

100

100

100

100

100

100

100

100

88

99

83

49

99

99

100

49

88

88

96

47

100

100

62

86

**APOPTOGENIC CASPASES**
