# Supplementary material for: Caspase-1 and IL-1β Processing in a Teleost Fish
Source: PLoS One. 2012 Nov 30;7(11):e50450. doi: 10.1371/journal.pone.0050450 (PMC3511578; doi:10.1371/journal.pone.0050450)
Supplement: Figure S4 — Alignment of IL-1β amino acid sequences from vertebrates belonging to different Classes. The regions having aspartate cleavage sites are shown (aspartates are shaded black). The sequences were aligned with CLUSTAL W [62] using the default parameters. GenBank accession numbers are shown within brackets. Aspartate residues are blue color and bold type. The caspase-1 cleavage site in mammalian IL-1β sequences is shaded yellow. The conserved aspartic acids homologous to the caspase-1 cleavage site in sea bass IL-1β sequence are shaded green. The conserved aspartic acids homologous to the putative caspase-1 cleavage site in avian IL-1β sequence are shaded red. Mutated aspartate residue in the sea bass and chicken IL-1β sequences are shaded black. (DOC) [file pone.0050450.s004.doc]

*Homo sapiens* IL-1β (NP_000567) … CPQTFQEN**D**LSTFFPFIFEEEPIFF**D**TW**D**NEA--YVH**D**AP---VRSLNCTLR**D**SQQKSL 134 … SQAENMPVFLGGTKGGQ**D**IT**D**FTMQFVSS----------------- 269

*Homo sapiens* IL-1β CRA c (EAW73607) … CPQTFQEN**D**LSTFFPFIFEEEPIFF**D**TW**D**NEA--YVH**D**AP---VRSLNCTLR**D**SQQKSL 168 … SQAENMPVFLGGTKGGQ**D**IT**D**FTMQFVSS----------------- 303

*Pan troglodytes* IL-1β (XP_515697) … CPQTFQEN**D**LSTFFPFIFEEEPIFF**D**TWENEA--YVH**D**AP---VRSLNCTLR**D**SQQKSL 134 … SQAENMPVFLGGTKGGQ**D**IT**D**FTMQFVSS----------------- 269

*Macaca mulatta* IL-1β (NP_001036221) … CPQIFQ**D**N**D**LSTLIPFIFEEEPVFL**D**TRNN**D**A--CVH**D**AP---VRSLHCTLR**D**AQLKSL 134 … SQAENMPVFLGGTRGGQ**D**IT**D**FTMQFVSS----------------- 269

*Macaca nemestrina* IL-1β (P51493) … CPQIFQ**D**N**D**LSTLIPFIFEEEPVFL**D**TRNN**D**A--CVH**D**AP---VRSLHCTLR**D**AQLKSL 134 … SQAENMPVFLGGTRGGQ**D**IT**D**FTMQFVSS----------------- 269

*Macaca fascicularis* IL-1β (P79182) … CPQIFQ**D**N**D**LSTLIPFIFEEEPVFL**D**TRNN**D**A--CVH**D**AP---VRSLHCTLR**D**AQLKSL 134 … SQAESMPVFLGGTRGGQ**D**IT**D**FTMQFVS------------------ 268

*Chlorocebus sabaeus* IL-1β (ACI28917) … CPQTFQ**D**N**D**LSTLIPFIFEEEPIFL**D**TSNN**D**A--CVH**D**AP---VRSLHCTLR**D**AQLKSL 134 … SQAENMPVFLGGTRGGQ**D**IT**D**FTMQFVSS----------------- 269

*Cercocebus torquatus atys* IL-1β (P46648) … CPQTFQ**D**N**D**LSTLIPFIFEEEPVFL**D**TCNN**D**A--CVH**D**AP---VRSLHCTLR**D**AQLKSL 134 … SQAENMPVFLGGTRGGQ**D**IT**D**FTMQFVSS----------------- 269

*Mus musculus* IL-1β (NP_032387) … FPWTFQ**D**E**D**MSTFFSFIFEEEPILC**D**SW**DDDD**NLLVC**D**VP---IRQLHYRLR**D**EQQKSL 135 … SQAEHKPVFLG-NNSGQ**D**II**D**FTMESVSS----------------- 269

*Rattus norvegicus* IL-1β (NP_113700) … CPWSFQ**D**E**D**PSTFFSFIFEEEPVLC**D**SW**DDDD**-LLVC**D**VP---IRQLHCRLR**D**EQQKCL 134 … SQAEHRPVFLG-NSNGR**D**IV**D**FTMEPVSS----------------- 268

*Meriones unguiculatus* IL-1β (BAD67162) … CPWTFQ**D**E**D**LKTFFSFIFEEEPILC**D**SWE**D**EQ--LVC**D**FP---IRQLHCRLR**D**EQQKCL 133 … SQAEHKPVFLG-SNSGQ**D**IV**D**FTLESVSS----------------- 267

*Sigmodon hispidus* IL-1β (AAL18817) … CPWTFQ**D**E**D**LRSFFSVIFEEEPIFC**D**YW**DD**EL--VVA**D**VP---VRQLHCRLR**D**EQQKCL 133 … SQAEHKPVFLG-NNSGQ**D**IV**D**FTMESVSS----------------- 267

*Cavia porcellus* IL-1β (Q9WVG1) … CTWAFQ**DDD**LRPLLPFIFEEEPIVC**D**TW**D**EEY---ES**D**TP---VPSRNCTLH**D**IQHKRL 132 … SQAEHKPVFLG-NNNGQ**D**II**D**FKLELVSS----------------- 266

*Capra hircus* IL-1β (ABI20514) … YEHVFR**DDD**LRSILSFIFEEEPVIFET-SS**D**--ELLC**D**AA---VQSVKCKLQ**D**REQKSL 131 … SQIEEKPVFLGHFRGGQ**D**IT**D**FRMETLSP----------------- 266

*Ovis aries* IL-1β (NP_001009465) … YEHVFR**DDD**LRSILSFIFEEEPVIFET-SS**D**--ELLC**D**AA---VQSVKCKLQ**D**REQKSL 131 … SQIEEKPVFLGRFRGGQ**D**IT**D**FRMETLSP----------------- 266

*Ovis canadensis* IL-1β (ABU94271) … YEHVFR**DDD**LRSILSFIFEEEPVIFET-SS**D**--ELLC**D**AA---VQSVKCKLQ**D**REQKSL 131 … SQIEEKPVFLGRFRGGQ**D**IT**D**FRMETLSP----------------- 266

*Bos taurus* IL-1β (ABX72065) … YAHVFH**DDD**LRSILSFIFEEEPVIFET-SS**D**--EFLC**D**AP---VQSIKCKLQ**D**REQKSL 131 … SQIEERPVFLGHFRGGQ**D**IT**D**FRMETLSP----------------- 266

*Bubalus carabanensis* IL-1β (Q2MH07) … YAHVFH**DDD**LRSILSFIFEEEPVIFET-SS**D**--EFLC**D**AP---VQSVKCKLQ**D**REQKSL 131 … SQIEERPVFLGHFRGGQ**D**IT**D**FRMETLSP----------------- 266

*Cervus elaphus* IL-1β (P51745) … YAHVFH**DDD**LRNVLSFIFEEEPVIFET-SS**D**--EFLC**D**AA---VQSVNCKLQ**D**REQNSL 131 … SHPEEKPVFLGHFRGGQ**D**IT**D**FRMETLSP----------------- 266

*Odocoileus virginianus* IL-1β (AAR91596) … YAHVFH**DDD**LRNILSFIFEEEPVIFET-SS**D**--EFLC**D**AA---VQSVNCKLQ**D**REQKSL 131 … SQTEEKPVFLGHFRGGQ**D**IT**D**FRMETLSP----------------- 266

*Sus scrofa* IL-1β (NP_001005149) … SSQVVC**DDD**PKSIFSSVFEEEPIVLEK-HAN--GFLC**D**ATP--VQSV**D**CKLQ**D**K**D**EKAL 132 … SQAEQKPVFLGNSKGRQ**D**IT**D**FTMEVLSP----------------- 267

*Lama glama* IL-1β (Q865X8) … YSQYFQ**DDD**LRNIFSLIFEEEPVTFET-CA**D**--**D**FVC**D**AV---VQSLYCKLQ**D**KEQKSM 132 … STAEQRPVFLGQSRGGQ**D**IT**D**FTMETLSP----------------- 267

*Tursiops truncatus* IL-1β (BAA87947) … CSQTFQ**DD**GLRSIFSLIFEEEPVIFET-Y**DD**--**D**LLC**D**AA---VQSLTCKLQ**D**R**D**QKSL 131 … SQAEEKPIFLGRSKGGH**D**IT**D**FTMEIISP----------------- 266

*Delphinapterus leucas* IL-1β (Q8WNR2) … CSQTFQ**DD**GLRSIFSLIFEEEPVIFET-Y**DD**--**D**LLC**D**AG---VQSLTCKLQ**D**R**D**QKSL 131 … SQAEEKPIFLGRSKGGH**D**IT**D**FTMEIISP----------------- 266

*Equus caballus* IL-1β (ABZ91976) … CSQAFQ**DDD**LRSLFSVIFEEEPIIC**D**NW**DDD**Y---VC**D**AA---VHSVNCRLR**D**IYHKSL 133 … SQAEKKPVFLGNTRGGR**D**IT**D**FIMEITSA----------------- 268

*Mustela putorius furo* IL-1β (A4UYK8) … CSLPLQG**DD**LMNVFHCIFEEEPIILEK-C**DD**N-AFVH**D**AP---PRSL**D**CKFQ**D**INQKSL 136 … SQAEAMPVFLGNNRGGH**D**IT**D**FTMELSS------------------ 270

*Phoca vitulina richardsi* IL-1β (Q6PUD2) … CSQPLQ**DDD**LKNVFCCIFEEEPIVCEV-Y**DDD**-AFVC**D**AP---LQSL**D**CKFR**D**KNQKSL 136 … SKAEAMPVFLGNTKGGQ**D**IT**D**FTMEFSS------------------ 270

*Eumetopias jubatus* IL-1β (Q6R2X3) … CSQPLQ**DDD**LKNIFCCIFEEEPIVCEV-Y**DDD**-AFVC**D**AP---LQSL**D**CKFR**D**ISQKSL 136 … SKAEAMPVFLGNTKGGQ**D**IT**D**FTMELSS------------------ 270

*Trichosurus vulpecula* IL-1β (Q9XS77) … SSQFFQ**D**N**D**LMNIFTNIFQEEPITFKNC**D**IYE----S**D**SSFRLVSSQ**D**CTIQ**D**INQKCL 138 … SQM**D**EQPVFLGNIRGGK**D**IT**D**FILA**D**F------------------- 269

*Ornithorhynchus anatinus* IL-1β (CAC80337) … YNTSFM**D**R**D**LM**D**IFTSIFKEEPISCSTWEQT---LVT**D**SLYHYLRCQEVTIW**D**EEHKSF 150 … SREE**D**EPVFLGASKGEEAITNFFLH--------------------- 290

***Gallus gallus IL-1β (NP_989855)*** ***… RSRDFADSDLSALLEEVFEPVTFQRLESSYAG-----APAFRYTRSQSFDIFDINQKCF 127 … SLQPRQPVGITNQPDQVNIATYKLSGR------------------- 267***

*Meleagris gallopavo* IL-1β (ABD49202) … TRR**D**FA**D**S**D**LSALLEEVFEPVTFQRLESSYAG-----APAFRYTRSQSF**D**IF**D**INQKCF 127 … SLQPRQPVGITNRP**D**QVNIATYKLSGR------------------- 267

*Columba livia* IL-1β (ABD49201) … AHK**D**FA**D**S**D**LG**D**FL**DD**IFEPVSFRRIESSYAG-----APVYRYTRSQSF**D**IL**D**IAQKCF 129 … SLQPRQPVGIT**D**KP**D**QVNIATYALSGH------------------- 269

*Anser anser* IL-1β (ABD49200) … RSR**D**FA**D**S**D**LSALLEEVFEPVTFQRLESSYAG-----APAFRYTRSQSF**D**IF**D**INQKCF 127 … SLQPRQPVGITNQP**D**QVNIATYKLSGR------------------- 267

*Taeniopygia guttata* IL-1β (XP_002195600) … SRK**D**FA**D**S**D**LGSFL**DD**IFEPVSFRCIRGSYTR-----APVFRYTRSQSF**D**IL**D**I**D**HKCF 129 … SLQPRQPVGITNTP**D**QVNIATYELSGR------------------- 269

*Xenopus laevis* IL-1β (CAC85480) … KERFFG**D**E**D**LLGLL**D**SIFVEEEIAFSQAKETH---ASASTYRYQRATTCRIK**D**TSNKCF 137 … SQRENELVQMVHQKNQEAIK**D**FNLFSVI------------------ 283

***Dicentrarchus labrax IL-1β (CAC41006)*** ***… MSTEFRDENLLNIMLESIVEEKIVFERGTTPTAQ-----YSKRR-EVQCSVTDSEKRSL 106 … AEENNRPVQMCQESAR-RHRAFNIDNLKVDPTTEDQVCPLLNGQ-- 261***

*Lateolabrax japonicus* IL-1β (ABP38359) … LGTEFR**D**ENLLNIML**D**NIVEEQIVFERSSTPP----A-QFTWTG-KELYNVT**D**SEKRSL 107 … AKQ**D**KKPVEMCLETAQ-RHRTFNL**D**LALEAASECQVSPS------- 258

*Sparus aurata* IL-1β (CAD11603) … RGTEFT**D**ENLLNILLESAVEERTVFER-TAKPAQ-----YTYNF-QSLYSVM**D**SEQRHL 105 … AEENNKPV**D**MCQESAR-RHRIFKFLPP--KPEVEGGEC-------- 253

*Pagrus major* IL-1β (AAP33156) … RSTEFT**D**ENLLNMLLESAVEERIVFER-TATPAQ-----YKYKF-QNLYSVM**D**SEQRHL 105 … AEENNKPVEMCQETTR-RHRIFKFLQP--NPEV**D**GGEC-------- 253

*Acanthopagrus latus* IL-1β (AAV74185) … RSTEFT**DD**NLI**D**MLLESAVEERTVFER-TAKPAQ-----YTYNF-QSPYSVM**D**SEQRHL 105 … AEENNKPV**D**MGQESAR-RHRIFKFLPP--KPAVG**D**GEC-------- 253

*Dentex dentex* IL-1β (CAD30552) … RSTEFT**D**ENLLNMLLESAVKERTVFER-TATPAQ-----YKYKF-QNLYSVM**D**SEQRHL 96 … ----------------------------------------------

*Diplodus puntazzo* IL-1β (CAD30551) … QSTEFT**DD**NLLNILLESAVKERNVFER-TAKPAQ-----YTYNF-QSLHSVM**D**SEQRHL 94 … ----------------------------------------------

*Epinephelus coioides* IL-1β (ABV02593) … LGTEFR**D**EHLLSIMLESIVEERNVFGCEATPPT**D**E-**D**-MITRTR-EY**D**CTVE**D**EEKRSL 109 … AEA**D**NMPVEMCQESTS-RYRAFTFS-AIKEETPTA----------- 254

*Oplegnathus fasciatus* IL-1β (ACH87392) … LSTEFR**D**EHLLNIMLESIVEEKVVFERSAAPPTQ-----FQQFG-EEQCTMA**D**TEKKSL 106 … AGENNKPVEMCLESAS-RHRTFTIEHQ---SST**D**CGSTCGSHVCPS 261

*Siniperca chuatsi* IL-1β (AAV65041) … RSTKCT**D**ENLLNFMMESIVEEQTVFERGSAPPLQ-----FRRT**D**-EYQCSVT**D**SKKRSL 104 … AV**D**NNKPVEMCLETAQ-RHRTFNIQHQ--SSNLPRAS--------- 251

*Latris lineata* IL-1β (ACQ99510) … LSTELR**D**ENLLNFMLESIVEEQIVFERSSAPPVQ-----FRCTG-V**D**QCSVT**D**SRKRSL 104 … TE**D**N-KPVEMCLESAQ-SYRTFSIQNLPRTTRMGCGSASGRQA--- 258

*Chionodraco hamatus* IL-1β (CAD92853) … LSTEFR**D**ENLLNFIM**D**SIVEEQIVFECASAPP----A-QITRT**D**-VEPQNIT**D**GEKRSL 104 … AEQNNKPVEMCLETAK-RFRSFNIG-**D**IQGNV**D**RQS---------- 250

*Rachycentron canadum* IL-1β (AAT65502) … LSTEFR**D**ENLINVMLENIMEEHVVLELGSAPPVQ-----FSRTG-EYQCSVT**D**SEQRSL 106 … AK**DD**NKPVEMCTET**D**N-RYRTFNIQRQS------------------ 246

*Tetraodon nigroviridis* IL-1β (CAE00572) … LSTEFR**D**ENLLNIMLETVVEEKIVLECYSAPPPQ---**D**KYSRRS-EISCSVT**D**SEKRSL 108 … AVE**D**NKPVEMCLESAL-RYTSFTIQPLKGNPSCEGEM--------- 257

*Scophthalmus maximus* IL-1β (CAC33867) … LGTEFR**D**ENLLSVMLESIVEEHIVFERSSSPP**D**Q-----FSRTG-VHRCSVT**D**EQKRNF 106 … SEQ**D**NKPVEMCQESAQ-RYQTFSIQRQI------------------ 246

*Paralichthys olivaceus* IL-1β (BAB86882) … LSTSFT**D**ENLLNIMMENIVXEHIVCERSSSPP**D**Q-----FSRRG-VYTCNIT**D**SQKRNF 106 … SEQ**D**NRPVMVGQKNAR-CYQTFNIQHQS------------------ 247

*Gadus morhua* IL-1β (CAD59733) … QSSEFR**D**E**D**LLNLLLENAL**D**EQLVLELTEAAPPR----GFTAIEPSQQCMLR**D**HQKRSM 114 … ATE**D**NLPVEVCLQSES-RYRSFTILQG------------------- 253

*Melanogrammus* *aeglefinus* IL-1β (CAD79352) … QSSEFR**D**E**D**LLNLLLENAL**D**EQLVLELTEATPPR----GFTAIEPSQQCMMR**D**IQKRSM 114 … ATE**D**NLPV**D**VCLQSAS-RYHSFTILQG------------------- 253

*Salmo salar* IL-1β (NP_001117054) … MGTEFK**D**K**D**LLNFLLESAVEEHIVLELESAPPTSRREAGFSSTS-QYECSVT**D**SENKCW 116 … QQ**D**NTKPV**D**MCQKAAPNRLTTFTIQRHN------------------ 260

*Oncorhynchus mykiss* IL-1β (CAC83518) … MGTEFK**D**K**D**LLNFLLESAVEEHIVLELESAPPASRRAAGFSSTS-QYECSVT**D**SENKCW 116 … QQ**D**YTKPV**D**MCQKAAPNRLTTFTIQRHN------------------ 260

*Oncorhynchus mykiss* IL-1β2 (CAB53541) … LG---R**D**EGLLNFLLESAVE---VLELESARTEASSRAAFSSKG-EYECSVT**D**SENKCW 110 … QQ**D**NTKMVNMCQRATLNRNTTFTIQRHN------------------ 254

*Salvelinus alpinus* IL-1β (CAJ29194) … MGTEFK**D**K**D**LLNFLL**D**SAVEEHIVLELESAPPASRRGAGFSSKS-QYECSVT**D**SENKCW 116 … QQ**D**-TKPV**D**MCQKAAPNRLTTFTIQRHN------------------ 259

*Conger myriaster* IL-1β (BAF73943) … RGTEFC**D**HELL**D**IMLENVFEESVTPVAV**D**TWRSKPN---VFYNTKVIEHSVC**D**Q**D**QKSL 107 … AVE**D**KRPVEMCKE**DD**SAHFTNFSFTHL------------------- 248

*Ictalurus punctatus* IL-1β (AAZ94731) … QSTEFT**D**QELFNVFI**D**NVIEESMVINLKC--TESKS---YSLQ**D**KVVRCTIC**D**KSKRAL 131 … SK**DDD**KPVQTCKQQ-SSHLQLFTLH**D**ETVVSQNEM----------- 280

*Ictalurus punctatus* IL-1β2 (AAZ94732) … QSTEFT**D**QELFNVFIENVIEESMVINLKC--TESKS---YSLQ**D**KVVRCTIC**D**KSKRAL 131 … SKE**D**YKPVQMCKQQ-SSHLQLFTLH**D**ETVVSQNEI----------- 280

*Cyprinus carpio* IL-1β (BAA24538) … SSGKFC**D**EELLGFILENVIEERLVKPL----NETPI---YSKTSLTLQCTIC**D**KYKKTM 131 … AF**DD**WEKVEMNQMP-TTRTTNFTLE**D**QKRI---------------- 276

*Cyprinus carpio* IL-1β2-1 (CAC19887) … SSGKFCQ**D**ALLNIILENVIEERLVKPL----NATQT---YCKTSRTLLCTVC**D**KYKKTL 129 … AF**DD**WKRVEMSQVP-T**D**RTT**D**FTLQ**D**-------------------- 272

*Cyprinus carpio* IL-1β2-2 (CAC19888) … SSGKFSQ**D**ALLNIILENVIEERLVKPL----NATQT---YRKTTRTLQCSVC**D**KYKKTL 129 … AF**DD**WKRVEMSQVP-T**D**RTT**D**FTLQ**D**-------------------- 272

*Carassius auratus* IL-1β1 (CAD12102) … SSGKFCE**D**ALLNFILENVIEERLVKPL----NVSQT---YSKSSRTLQCTIC**D**KYKKTL 129 … AF**DD**WERVEMIQVP-T**D**RTTNFTLE**D**-------------------- 272

*Carassius auratus* IL-1β2 (CAD12103) … APGKICE**D**ALLSFFLENVIEERMVN-------VAPT---YNKTRQTLQCTVC**D**KYKKTL 90 … AF**DD**WEKVEMYQRP-TERIP**D**FTLE**D**QKLIRT-------------- 238

*Danio rerio* IL-1β (AAH98597) … ST-EFGEKEVL**D**MLMANVIQEREVNVV----**D**SVPS---YTKTKNVLQCTIC**D**QYKKSL 128 … AYE**D**SQMVEM**D**RK**D**-TERIINFELQ**D**KVRI---------------- 272

*Scyliorhinus canicula* IL-1β (CAC09435) … **D**GPSLT**D**A**D**LLGNF**D**ALLE**D**AITCTSYGEVEQ----AACSFRFMRSEQEHMT**DD**Q**D**RSL 167 … SRRNRQPIEL**D**EKKNHKRITIFTA**D**--------------------- 301

*Triakis scyllium* IL-1β (BAC76440) … **D**GAPFA**D**T**D**LLVNF**D**ALLEEAVTCTSY**DD**VEM----AVCSYRFMASERQQMK**DD**R**D**QSL 168 … SRKNRQPVEL**D**VKENHKRITIFTS**D**--------------------- 302
