# Supplementary material for: Caspase-1 and IL-1β Processing in a Teleost Fish
Source: PLoS One. 2012 Nov 30;7(11):e50450. doi: 10.1371/journal.pone.0050450 (PMC3511578; doi:10.1371/journal.pone.0050450)
Supplement: Table S1 — Primer sequences and application. (DOC) [file pone.0050450.s006.doc]

**Table SI.** Primer sequences and application.

| Primer | Sequence^a^ | Application |
| --- | --- | --- |
| APv | 5’ – GGCCACGCGTCGACTAGTAC(T_17_)V – 3’ | cDNA synthesis |
| AUAP | 5’ – GGCCACGCGTCGACTAGTAC – 3’ | cDNA amplification |
| CASP1FW1 | 5’ – GARAARGAYGARGARAAYATG – 3’ | cDNA amplification |
| CASP1RV1 | 5’ – GACYTTNCKRAANARYTCYTC – 3’ | cDNA amplification |
| CASP1RV2 | 5’ – GCCRTGNSWCATDATNACNAC – 3’ | cDNA amplification |
| DLCASP1FW7 | 5’ – TCTGAACCAGAGCGCATTAC – 3’ | 5’- Racing |
| DLCASP1RV1 | 5’ – CATATCCCAGAGCTTTGAGAAGT – 3’ | 5’- Racing |
| DLCASP1RV2 | 5’ – CTCAATCATGGCAGCGTCAA – 3’ | 5’- Racing |
| DLCASP1RV3 | 5’ – TGTCAGTCTCCCTGAGTTTC – 3’ | 5’- Racing |
| DLCASP1RV5 | 5’ – CTTTCAGGAATCGTGTGGCC – 3’ | 5’- Racing |
| DLCASP1FWEcoRI | 5’ – CGGAATTCTCTGAACCAGAGCGCATTA – 3’ | Genomic amplification |
| DLCASP1RVEcoRI | 5’ – CCGAATTCCGACAGATGGAGCAAACAGTGTG – 3’ | Genomic amplification |
| DLCASP1FW4 | 5’ – TGTGGCCCTTCTCATCACTA – 3’ | Probe amplification |
| DLCASP1RV6 | 5’ – GGCCTGGATGATGATGATCT – 3’ | Probe amplification |
| DLCASP1FW11 | 5’ – AGAAATGGCAGATAAGGAG – 3’ | Expression |
| DLCASP1RV16 | 5’ – GAGGACAGTTTGCCCTTAGA – 3’ | Expression |
| DLCASP1RV17 | 5’ – CGGAGTGCAAGTTGCAATGT – 3’ | Expression |
| DLCASP1RV18 | 5’ – AGCGTTGCATGACTGTTCTG – 3’ | Expression |
| DLCASP1RV19 | 5’ – AAAGCGTTGCATGACCGGGG – 3’ | Expression |
| DLCASP1FWNdeI | 5’ – GGAATTCCATATGGCAGGTAAGGAGCTTTTCAG – 3’ | Recombinant production |
| DLCASP1RVXhoI | 5’ – CCGCTCGAGGCCTGGGAAGAAGTAGAA – 3’ | Recombinant production |
| DLISO2/3RVXhoI | 5’ – CTCGAGAAGCGTTGCATGAC – 3’ | Recombinant production |
| DLISO4RVXhoI | 5’ – ACTCGAGACGGAGTGCAAGT – 3’ | Recombinant production |
| DLIL1βFWNde3 | 5’ – AGCAGCATATGGAATCTGAGATGAA – 3’ | Recombinant/*in vitro* translation |
| DLIL1βRVXho4 | 5’ – CCGCTCGAGCTGTCCATTCAAAAGGGG – 3’ | Recombinant/*in vitro* translation |
| DLIL1βFWNde4 | 5’ – CATATGAGCGAAAAGAGGAGCTTAGTTCTG – 3’ | Recombinant/*in vitro* translation |
| DLIL1βRVXho1 | 5’ – GCGCTCGAGTTACTGTCCATTCAAAAGGGGACAA– 3’ | Recombinant/*in vitro* translation |
| DLIL1βMUT0FW1 | 5’ – CCCCTTTTGAATGGACAGTGAGAGCACCACCACCACC – 3’ | Site direct mutagenesis (Remove LE6His) |
| DLIL1βMUT0RV1 | 5’ – GGTGGTGGTGGTGCTCTCACTGTCCATTCAAAAGGGG – 3’ | Site direct mutagenesis (Remove LE6His) |
| DLIL1βD^60^FW1 | 5’ – GATGAGCACTGAGTTCAGAGCGGAAAACCTGCTA – 3’ | Site direct mutagenesis (D^60^A) |
| DLIL1βD^60^RV1 | 5’ – TAGCAGGTTTTCCGCTCTGAACTCAGTGCTCATC – 3’ | Site direct mutagenesis (D^60^A) |
| DLIL1βD^100^FW1 | 5’ – AATGCAGCGTGACCGCGAGCGAAAAGAGGA – 3’ | Site direct mutagenesis (D^100^A) |
| DLIL1βD^100^RV1 | 5’ – TCCTCTTTTCGCTCGCGGTCACGCTGCATT – 3’ | Site direct mutagenesis (D^100^A) |
| DLIL1βD^252^FW1 | 5’ – TCCAACAACTGAGGCGCAAGTTTGTCCC – 3’ | Site direct mutagenesis (D^252^A) |
| DLIL1βD^252^RV1 | 5’ – GGGACAAACTTGCGCCTCAGTTGTTGGA – 3’ | Site direct mutagenesis (D^252^A) |
| HSIL1βD^116^FW1 | 5’ – GAGGCTTATGTGCACGCGGCACCTGTACGA – 3’ | Site direct mutagenesis (D^116^A) |
| HSIL1βD^116^RV1 | 5’ – TCGTACAGGTGCCGCGTGCACATAAGCCTC – 3’ | Site direct mutagenesis (D^116^A) |
| HSIL1βD^128^FW1 | 5’ – ACTGCACGCTCCGGGCTTCACAGCAAAAAA – 3’ | Site direct mutagenesis (D^128^A) |
| HSIL1βD^128^RV1 | 5’ – TTTTTTGCTGTGAAGCCCGGAGCGTGCAGT – 3’ | Site direct mutagenesis (D^128^A) |
| HSIL1βmat1FWNcoI | 5’ – CCCCATGGCACCTGTACGATCACTGAACT – 3’ | *In vitro* translation |
| HSIL1βmat1_2RVXhoI | 5’ – CGGCTCGAGTCAGGAAGACACAAATTGCAT – 3’ | *In vitro* translation |
| HSIL1βmat2FWNcoI | 5’ – GACCATGGCGTCACAGCAAAAAAGC – 3’ | *In vitro* translation |
| GGIL1βFWNdeI | 5’ – GGCATATGGCGTTCGTTCCCGACCTGGAC – 3’ | *In vitro* translation |
| GGIL1βRVXhoI | 5’ – TTCTCGAGTCAGCGCCCACTTAGCTTGTAGGT – 3’ | *In vitro* translation |
| GGIL1βD^77^FW | 5’ – GAGGCCGAGGAGCAGGGCATTTGCTGACAGCGA – 3’ | Site direct mutagenesis (D^77^A) |
| GGIL1βD^77^RV | 5’ – TCGCTGTCAGCAAATGCCCTGCTCCTCGGCCTC – 3’ | Site direct mutagenesis (D^77^A) |
| GGIL1βD^80^FW | 5’ – GGGACTTTGCTGCGAGCGACCTGAG – 3’ | Site direct mutagenesis (D^80^A) |
| GGIL1βD^80^RV | 5’ – CTCAGGTCGCTCGCAGCAAAGTCCC – 3’ | Site direct mutagenesis (D^80^A) |
| GGIL1βD^82^FW | 5’ – AGGGACTTTGCTGACAGCGCACTGAG – 3’ | Site direct mutagenesis (D^82^A) |
| GGIL1βD^82^RV | 5’ – CTCAGTGCGCTGTCAGCAAAGTCCCT – 3’ | Site direct mutagenesis (D^82^A) |
| GGIL1βmat1FWNdeI | 5’ – GGCATATGATCTTCGACATCAACCAGAAGTG – 3’ | *In vitro* translation |
| GGIL1βmat2FWNdeI | 5’ – GGCATATGATCAACCAGAAGTGCT – 3’ | *In vitro* translation |
| GGIL1βmat4FWNdeI | 5’ – GGCATATGAGCGACCTGAGCG – 3’ | *In vitro* translation |

^a^ R= A/G; Y= C/T; N= A/C/T/G; K= G/T; D=A/G/T; W=A/T; S=G/C ; V=A/C/G
