# Supplementary material for: Caspase-1 and IL-1β Processing in a Teleost Fish
Source: PLoS One. 2012 Nov 30;7(11):e50450. doi: 10.1371/journal.pone.0050450 (PMC3511578; doi:10.1371/journal.pone.0050450)
Supplement: Table S2 — Amino acid sequence conservation for caspase-1 of different species. (DOC) [file pone.0050450.s007.doc]

**Table SII.** Amino acid sequence conservation for caspase-1 of different species.

| Species | ***Dicentrarchus labrax*** | | | Accession number |
| --- | --- | --- | --- | --- |
|  | Identity | Similarity | Gaps |  |
| *Sparus aurata* | 62.7% | 76.7% | 4.7% | CAM32183 |
| *Danio rerio* caspy | 38.5% | 53.4% | 19.7% | NP_571580 |
| *Danio rerio* caspy2 | 30.7% | 45.0% | 23.8% | NP_690840 |
| *Rattus norvegicus* | 37.0% | 52.3% | 10.0% | NP_036894 |
| *Mus musculus* | 37.0% | 51.2% | 12.1% | NP_033937 |
| *Xenopus laevis* XICE-a | 36.9% | 51.1% | 9.3% | BAA14017 |
| *Homo sapiens* isoform alpha percursor | 36.0% | 51.1% | 8.6% | NP_150634 |
| *Felis catus* | 35.4% | 51.6% | 11.3% | NP_001009365 |
| *Gallus gallus* | 26.4% | 39.1% | 31.6% | AAC69917 |

The percentages of similarity and identity were calculated by pair-wise alignments with the program needle (Needleman & Wunsch (1970). J Mol Biol 48(3):443-453) as implemented at EBI web site (www.ebi.ac.uk) with first and extending gap penalties of 10 and 0.5, respectively.
